# Supplementary material for: Advising parents when their child has a fever: a phenomenographic analysis of nurses’ perceptions when working at a telephone helpline, at primary care or at a paediatric emergency department in Sweden
Source: BMJ Open. 2024 Jan 29;14(1):e074823. doi: 10.1136/bmjopen-2023-074823 (PMC10826557; doi:10.1136/bmjopen-2023-074823)
Supplement: Supplementary data [file bmjopen-2023-074823supp001.pdf]

Interview guide

Background questions

- Which workplace do you have?
- How long have you worked there?
- What education do you have?
- How old are you?

Interview questions

1. **What is advising parents whose child has a fever for you?**

- Tell us how it is done when you give advice to parents whose child has a fever?
- What experiences do you have of your work in advising parents whose children have a fever? Examples of situations perceived as positive/ difficult.
- How do you perceive that you receive support/can be a support to parents whose children have a fever?
- How do you perceive the parents' need for counseling when the child has a fever?
- What opportunities do you see with your consultancy?

Follow up questions like; can you tell me more, what do you mean when you say?
