# Supplementary material for: Advising parents when their child has a fever: a phenomenographic analysis of nurses’ perceptions when working at a telephone helpline, at primary care or at a paediatric emergency department in Sweden
Source: BMJ Open. 2024 Jan 29;14(1):e074823. doi: 10.1136/bmjopen-2023-074823 (PMC10826557; doi:10.1136/bmjopen-2023-074823)
Supplement: Author's manuscript [file bmjopen-2023-074823.draft_revisions.pdf]

BMJ Open

BMJ Open is committed to open peer review. As part of this commitment we make the peer review history of every article we publish publicly available.

When an article is published we post the peer reviewers' comments and the authors' responses online. We also post the versions of the paper that were used during peer review. These are the versions that the peer review comments apply to.

The versions of the paper that follow are the versions that were submitted during the peer review process. They are not the versions of record or the final published versions. They should not be cited or distributed as the published version of this manuscript.

BMJ Open is an open access journal and the full, final, typeset and author-corrected version of record of the manuscript is available on our site with no access controls, subscription charges or pay-per-view fees (http://bmjopen.bmj.com).

If you have any questions on BMJ Open's open peer review process please email info.bmjopen@bmj.com

BMJ Open

Advising parents when their child has a fever – a phenomenographic analysis of nurse's perceptions

Journal:	*BMJ Open*
Manuscript ID	bmjopen-2023-074823
Article Type:	Original research
Date Submitted by the Author:	18-Apr-2023

Complete List of Authors:	Westin, Emma; Linnaeus University - Vaxjo Campus, Department of Health and Caring Sciences ; Department of pediatrics, Växjö Central Hospital, Region Kronoberg<br>Gustafsson, Ingrid; University College of Boras Faculty of Caring Science Work Life and Social Welfare, Department of caring sciences; Linnaeus University Faculty of Health and Life Sciences, Department of health and caring sciences<br>Svensson, Anders; Linnaeus University Faculty of Health and Life Sciences, Health and Caring Science; Region Kronoberg<br>Sund, M; Linkopings universitet,<br>Elmqvist, Carina; Linnaeus University Faculty of Health and Life Sciences, Dept. of Health and Caring Sciences, Linnaeus University; Linnaeus University Faculty of Health and Life Sciences, Centre of Interprofessional Cooperation within Emergency care (CICE)

Keywords:	Nursing Care, PAEDIATRICS, QUALITATIVE RESEARCH

SCHOLARONE™
Manuscripts

I, the Submitting Author has the right to grant and does grant on behalf of all authors of the Work (as defined in the below author licence), an exclusive licence and/or a non-exclusive licence for contributions from authors who are: i) UK Crown employees; ii) where BMJ has agreed a CC-BY licence shall apply, and/or iii) in accordance with the terms applicable for US Federal Government officers or employees acting as part of their official duties; on a worldwide, perpetual, irrevocable, royalty-free basis to BMJ Publishing Group Ltd ("BMJ") its licensees and where the relevant Journal is co-owned by BMJ to the co-owners of the Journal, to publish the Work in this journal and any other BMJ products and to exploit all rights, as set out in our licence.

The Submitting Author accepts and understands that any supply made under these terms is made by BMJ to the Submitting Author unless you are acting as an employee on behalf of your employer or a postgraduate student of an affiliated institution which is paying any applicable article publishing charge ("APC") for Open Access articles. Where the Submitting Author wishes to make the Work available on an Open Access basis (and intends to pay the relevant APC), the terms of reuse of such Open Access shall be governed by a Creative Commons licence – details of these licences and which Creative Commons licence will apply to this Work are set out in our licence referred to above.

Other than as permitted in any relevant BMJ Author's Self Archiving Policies, I confirm this Work has not been accepted for publication elsewhere, is not being considered for publication elsewhere and does not duplicate material already published. I confirm all authors consent to publication of this Work and authorise the granting of this licence.

For peer review only

Advising parents when their child has a fever
# – a phenomenographic analysis of nurses' perceptions

Abstract

Objectives: To describe nurses' perceptions of advising parents when their child has a fever.

Design: inductive, descriptive study with a qualitative approach was conducted i.e. phenomenography which describes the different perceptions that exist of a phenomenon.

Setting: Participants were recruited from three different parts of the healthcare system from four regions in the south of Sweden: the telephone helpline 1177 Vårdguiden; primary healthcare, including child healthcare; and a pediatric emergency department.

Participants: A strategic sample was used. To be included, the participants had to have experience advising parents of febrile children between birth and five years of age. A total of 24 participants were interviewed.

Results: The nurses described advising parents when their child has a fever as four different kinds of balancing acts: Balancing between the parents' story and objective assessment, balancing between listening and teaching, balancing between self-confidence and trust in the expert, and balancing between independence and having someone by one's side.

Conclusions: Giving advice to parents when a child has a fever is a process where the nurse needs to listen, assess and give advice dependent on the situation. This is perceived as a balancing act where the nurse must make a correct assessment that depends on the parents' story. Creating a trusting relationship is perceived as necessary for parents to assimilate the advice that is provided. Surprisingly, what dominates are the nurses' perceptions of the inner qualities required to achieve a balance in the process. This shows the importance of experience and support from colleagues.

Strengths and limitations of this study

- Two of the five authors have experience in the phenomenographic method, and all of the authors have experience in qualitative methods.
- The first author carried out and transcribed all the interviews.
- There is great variation in the participants workplace, work experience and age.
- The interviews were done via video-conference call, and although most of the participants were accustomed to that way of communicating, this could limit the participants' ways of expressing themselves.

Introduction

Feverish children cause concerns and are among the most common reasons why parents contact telephone nursing services (1-3), primary care (4-5) or children's emergency departments (5-9). The most common expectations parents have are to get their child a physical examination by a physician, and to receive reassurance and information about warning signs (10).

The initial meeting within the healthcare system is usually with a nurse, who is responsible both for creating a trusting relationship and for providing adequate information (11). According to Halldórsdóttir (1996), this first encounter is decisive in whether the meeting and the relationship will be perceived as caring or non-caring (12). It is important that the nurses can identify the parents' needs, which can vary depending on background, expectations and previous experience, in order to decide what strategies that can be used to empower them (13).

In Sweden, parents can generally seek advice on their child's fever via telephone nursing services or within primary care (14). The telephone helpline 1177 Vårdguiden (henceforth 1177) are accessible around the clock all year round. The main focus for the telenurses at 1177 is to advise on different health problems (15). To support their advice, they use a computerized decision support tool (CDST); the same system is also frequently used by nurses who work in primary care. Primary care centers are responsible for preventing, diagnosing and treating illnesses, and, if necessary, sending referrals to specialist care (16). They must offer services for both planned and unplanned visits in general medicine during the day, as well as some on-call services. Primary care also includes child healthcare (17). The goal of child healthcare is to promote health and prevent illness in children from birth until the child starts school by following the child's development and health status and providing support to parents (18). If the child has acute symptoms, the parents should go directly to the pediatric emergency department, whose main purpose is to provide fast and highly specialized care for acute, sometimes life-threatening conditions. However, it is common for parents to go there for minor illnesses; in those cases, advice from a nurse may be the only course of action (7).

Previous studies have examined parents' views of having a child with fever, with or without contact with the healthcare system (19, 20). Less is known about nurses' perceptions of advising these parents. In this study, advising is defined as helping the parents assess the need for healthcare, give advice and information about measures that the parents can do themselves, and, if necessary, refer to appropriate level of care (21). Knowledge about nurses' perceptions is needed in order to train and support nurses taking on this role. This study therefore aims to describe nurses' perceptions of advising parents when their child has a fever, in a Swedish context.

Methods

For peer review only

Design

inductive, descriptive study with a qualitative approach was conducted i.e. phenomenography which describes the different perceptions that exist of a phenomenon. This is referred to by Marton (22) as the second-order perspective. The first-order perspective concerns objective observations, i.e., facts (22). Perceptions, on the other hand, are unspoken, implied and unreflected. It is perceptions that form the frame of reference for our thinking and the basis for our opinions (23).

Participants

A strategic sample was used that included nurses from three different parts of the healthcare system and from four different regions in southern Sweden, to obtain a rich and varied picture of the phenomenon. To be included, the nurses had to have experience advising parents of febrile children between birth and five years of age. The nurses were informed by the department head at each workplace. Those who chose to participate contacted the author directly. The participants received both written and oral information about the study and provided informed written consent. They were informed that participation was voluntary and that they had the right to cancel their participation in the study at any time. A total of 24 participants were included, evenly distributed between the different parts of the healthcare system and different regions of Sweden. The participants varied between 27 and 64 years (average 45), and their experience varied from 11 months to 25 years. Of the 24 nurses, 13 had at least one specialization, most commonly pediatric nurse or primary healthcare nurse (see Table 1).

Table 1. Demographics of 24 nurses included in the study.

	1177 (n=8)	Primary health care central and child health central (n=8)	Pediatric emergency department (n=8)
Gender (F/M)	8/0	8/0	6/2
Specialist nurse education (n)	2	7	4
Mean work experience (years)	5.6	8.6	7.8

Data collection

For peer review only

The interviews were conducted via Zoom by the first author during the period from March 2021 to October 2021. Data were collected through semi-structured interviews. The interviews began with an open-ended question: "What is advising parents whose child has a fever for you?" Follow-up questions such as "Can you tell me more?" and "What do you mean when you say…?" were used in order for the participants to reflect on the phenomenon and deepen their reasoning. Two pilot interviews were conducted, both of which were included in the study. The interviews lasted between 22 and 51 minutes (average 37 minutes) and were transcribed verbatim.

Data analysis

Data analysis was performed following the seven-step model of Larsson and Holmström (2009) (see Box 1). The analysis is not linear, but is rather a back and forth motion between the whole and the parts (24). For an overview of dominant (++) and non-dominant (+) ways of understanding nurses' perceptions of advising parents when their child has a fever, see Table 2.

Box 1. The seven steps of data analysis, according to Larsson and Holmström (2009).

1. Read the whole text from the transcribed interviews.
2. Read again and mark where the nurses gave answers to the interview questions.
3. In the marked passages, look for the focus of the attention of the nurses and how they describe their way of giving advice. Make a preliminary description of each nurse's predominant way of understanding their work.
4. Based on similarities and differences, group the descriptions into categories.
5. Search out non-dominant ways of understanding.
6. Structure the outcome space.
7. Assign each category of description a metaphor (in this case, each category came out as a balance).

Table 2. Overview of dominant (++) and non-dominant (+) ways of understanding nurses' perceptions of advising parents when their child has a fever.

For peer review only

Interview	The parents' story and objective assessment	Listening and teaching	Self-confidence and trust in the expert	Independence and having someone by one's side
1	+	+	++	++
2	+	++	++	++
3	+	+	+	++
4	++	+	++	++
5	++	++		+
6	++	++	++	++
7	+	++	+	++
8	++	++	+	++
9	+	++	++	++
10	+	+	+	++
11	+	+	+	+
12	++	+	+	++
13	+	+	+	++
14	+	++	++	++
15	++	+	+	++
16	+	+	++	++
17	++	++	+	++
18	+	+	+	++
19	+	+	+	++
20		+	+	++
21	+		+	+
22	+	+	+	++
23		+	+	+
24	+		+	++

Ethical considerations

Ethical approval was granted by the Swedish Ethical Review Authority (Dnr: 2020-03731). In accordance with the Declaration of Helsinki (25), the integrity and dignity of the participants have been respected in that their data and interviews were treated confidentially (25).

Results

The nurses described advising parents when their child has a fever as four different kinds of balancing acts: Balancing between the parents' story and objective assessment, balancing between listening and teaching, balancing between self-confidence and trust in the expert, and balancing between independence and having someone by one's side. All these balancing acts are part of and influence the process of giving advice, but one of them are expressed more dominantly than the others. For a schematic view of the outcome space, see Figure 1.

Balancing between the parents' story and objective assessment

For peer review only

The nurses perceive advising on fever as a balance between the parents' story and an objective assessment. Not seeing the sick child, as with telenursing, or when the nurse and parent don't speak the same language, is perceived as an additional difficulty. The nurse's objective assessment is based on the parents' subjective interpretation of the child's symptoms and their ability to describe them.

“There, we are also dependent on the parents to actually be able to make an assessment, so they may be dependent on us to get answers, but I am just as dependent on their answers to be able to make my assessment // it's quite interesting actually that it's not just them who need us, but we actually need them, and they know their child best". Q17

Nurses are dependent on the parents’ stories, but the parents’ worries may cause them to assess their children as sicker than they are; at the same time, parents know their children best and their concerns should be taken seriously. This interaction is perceived as a collaboration between nurse and parent where the expertise of the parents concerning their children needs to be respected. Parents need to provide enough information about the wellbeing of their children for nurses to be able to make an assessment, which requires good communication skills. Worry is perceived as potentially blocking this knowledge and affect the ability of parents to accurately assess their child’s fever. The nurses’ perceptions are that the degree of worry can be influenced by several different factors such as number of children, personality, culture, resources and previous experiences. Some parents have experienced a serious event in connection with a fever; others have read scary stories that are shared in the media and on the internet. Parents from countries where serious infections with fever are more common were perceived by the nurses as having greater worry about fever.

"The difficult thing is that there is a lot, there can be a lot of worry and then you don't really get the facts, but it is the worries that comes out". J10

Balancing between listening and teaching

The nurses in this study perceived advising about fever as a balance between listening and teaching. Providing information about fever is considered part of a nurse’s job, and the nurses perceived that the interaction between parents and themselves plays a role in how advice is received. It’s not just about teaching general knowledge in case of fever, which is something that is simple in and of itself, but the nurse has to listen to the needs of parents and start from there in order to address their specific concerns.

“It will be to create a trust and then to make a knowledge inventory and then come along, with the information, or the advice that I want to give. If I start by giving advice, I don't think the family sees themselves being seen, then it becomes more dismissive even though it is well-intentioned on my part that I want to help. So it has to happen in the right order there” H8

For peer review only

When time is constrained, advising may become more standardized and less based on individual needs. This is perceived as creating a feeling in the parents that they are not being taken seriously. The nurses also perceive that sometimes consensus regarding what is classified as a serious illness is lacking, but regardless of what their own assessment is, the parents' concerns need to be confirmed so they feel they are being heard. This includes, for example, booking an appointment to see a doctor even if the nurse assesses this as being unnecessary. At the same time, the nurses perceive that the needs and wishes of the parents must be weighed against the resources of the healthcare system. The nurses' perceptions are that worried parents have the right to seek medical care, but there are benefits to consensus when it comes to, for example, the waiting time at the children's emergency department. In the absence of consensus, the likelihood of parents following such advice is perceived as reduced. In these cases, it is considered likely that the parents will seek additional advice, either at the same place or elsewhere. Taking the parents seriously, confirming their concerns and explaining the basis for the assessment are perceived as tools for achieving consensus.

"It's important not to diminish their worries but to show that you listen to them and that it's okay that they're worried. But you have to explain to them, if it's the case that we're not worried, why not in that case so that they understand our work". E6

Balancing between self-confidence and trust in the expert

The nurses' perceptions are that advising on fever is a balance between self-confidence and trust in the expert. Nurses at the children's emergency department are perceived as having the most expertise advising parents, most often using their own knowledge and local directions as guidance. Nurses in other parts of the healthcare system often described using a computerized decision support tool, CDST. It is considered evidence-based support, mainly for nurses who lack experience, but it is also perceived as requiring some experience to be interpreted correctly. CDST provides a structure and has clear directives when it comes to fever. At the same time, it can be perceived as undifferentiated and limited in more complex cases, and in such cases some nurses place more trust in their own knowledge. The fact that some nurses use CDST and some don't may result in parents receiving different answers depending on where they seek advice. This is perceived as causing confusion and sometimes resulting in contradictions between nurses in different parts of the healthcare system.

"Those who have done this decision support, they are specialists, so it's not someone sitting at home and tinkering with this // then, as I said, you have to have your own experience with you, also as a nurse, a newbie, newly trained nurse would not have been so appropriate perhaps to put... in advising, it is not, it is not so simple, so it is not". N14

The nurses perceive though, that the parents generally trust their advice, even if stories about when care has failed to detect and treat serious illness can reduce trust. The trustworthiness is perceived to increase if several people (or sources) say the same thing, if the assessment involves a physician or if the nurse and the parent have an established relation. Establishing

trust via phone poses a greater challenge than meeting someone in person. The nurses perceive themselves as providing the most reliable and evidence-based advice and that parents see them as experts and often trust them over themselves.

"There is no opinion in that [the advice], but that it is actually a science that we rely on, I think it is a support that we are actually nurses that they talk to, it's not just anyone, like a grandmother or their mother" T20

Parents are perceived as having different abilities with regard to seeking knowledge about their child's fever, but it's generally perceived as being difficult to sift through all the available information and assess what is reliable. The nurses perceive that this information overload makes it difficult for parents to fully trust their own knowledge or that of their relatives. The nurses also expressed that even if the parents have general knowledge of fever, they may need help to assess the child's condition and require confirmation that their knowledge is being applied correctly to the situation.

Balancing between independence and having someone by one's side

The nurses perceive that advising on fever is a balance between independence and having someone by one's side. Both training and experience are required, especially on the phone, because fever can represent both harmless and serious conditions. This is also perceived as involving great responsibility as it requires caring for parents as well as the child. The nurses need to feel secure in their professional role and not be distressed by the parents' worries. Independently assessing a child with a fever can be a challenge, especially if it involves a younger child. With increasing experience and knowledge comes independence, but it is still important to have access to support, to have someone by one's side.

"But I think I have the greatest support of my colleagues, so partly I think, despite having worked for a long time, I think it's quite nice that one of my colleagues, it could be the assistant nurse, has also seen the child and says the same as me, that I'm not worried. They have, after all, are often very experienced, have seen a lot of children. Then I feel even more confident in my assessment and can provide better support". B2

This is also perceived by the nurses as being true for the parents. Parents today, though, are perceived as being fearful of fever, of lacking knowledge and support from their relatives, and instead need to seek this support from the healthcare system. This is considered to be one of the most common reasons for parents to seek advice. Therefore, the nurses perceive that parents need and expect healthcare to be accessible. Providing a plan for how parents should deal with their children having a fever and where to turn when this occurs is perceived by the nurses as a way of increasing the independence of parents, by giving them tools to take control of their situation. This can be combined with a follow-up call, where the nurse can determine whether the parents followed the advice that was given and can evaluate the results. This is perceived by the nurses as them supporting the parents.

For peer review only

"Because then I show that I actually care if the situation has improved or not, if they get the help they need, that someone cares about them and that they are not standing there alone in case something happens". D4

Discussion

This study describes nurses' perceptions of advising parents when their child has a fever as four different but related balancing acts. Listening to the parents, making assessments and offering advice can be considered the foundation of the advice-giving process, but the nurses' perceptions show that this is less dominant than the category containing personal characteristics and support. Overall, these results align with those of Greenberg (26), who has previously described telephone nursing, however as a process comprising three phases. Greenberg's (26) process also starts with listening to the caller's story and gathering information. In the second phase, the nurse assesses the problem and determines the proper interventions, sometimes with the support of a colleague. The third phase is called output and consists of different nursing actions such as referring to primary care or offering advice for self-management, reassurance and validation. This also describes personal characteristics of the nurse, for example, experience, as influencing factors of the telenursing process (26).

The nurses in the present study described difficulties in balancing between the parents' stories and an objective assessment sometimes as a result of difficulties in communication. Earlier studies show that parents often assess and express symptoms differently than healthcare personnel. For example, parents often start from the view of a child's normal behavior and judge the health of the child in relation to how much the behavior deviates from the usual (27-29). Parents may find it difficult to assess and express specific symptoms such as dehydration and breathing problems if they have not experienced them before (29). Conversely, healthcare personnel usually assess illness based on specific symptoms, which can lead to mistakes in communication (30). Greenberg (26) emphasizes that nurses interpreting between healthcare information and information that the caller can understand serves as an important component that links together different aspects of the telenursing process (26). The nurses in the present study perceived that parents' worry affects the way parents assess the symptoms of their children. Previous studies show that it can be difficult for parents to assess their sick child and know when it is time to seek medical care or when self-care is enough (31-32). Nevertheless, the present results show that nurses understand the importance of listening to parents' feelings about and their view of the child's illness. This is confirmed by studies that show a connection between self-rated parental worry and hospitalization (33-34).

The nurses in the present study perceived that there had to be a balance between listening and teaching and that their relationship with the parents would decide whether they could reach them with their advice. Halldórsdóttir (12) argues that in order to achieve a caring relationship, the nurse must appear to genuinely care. The nurse needs to build a bridge where the parent feels a sense of belonging in the meeting. If, on the other hand, the parent feels that the nurse is lacking in caring and doesn't want or can't meet, a wall is built that makes it difficult to communicate and create trust (12). For example, the nurses in the present study

For peer review only

described a gatekeeping role; even though the nurses wanted to provide service, they felt limited by the resources of the healthcare system. Being refused access to the desired healthcare can give a feeling of not being taken seriously (28, 33), which can then build a wall between parents and nurses. If there is a disagreement about how serious the child's illness is and the nurse fails to explain her assessment, it can lead to mistrust of the given advice. If the parents are not satisfied with the advice, there is a risk that they will seek advice again in the near future (35-36). To some extent, this may be due to the parents failing to regain a sense of control over the situation (27, 33).

The nurses in the present study described a perceived hierarchy within the healthcare system with regard to who was considered to be experts. This aligns with what previous studies have shown. A common reason for parents turning to the pediatric emergency department for a minor illness is that they have a higher level of trust in specialist care (often in combination with poor availability in primary care) (8-10, 37-38). However, it has also been shown that within the healthcare system there is a large proportion of incorrect referrals to the pediatric emergency department (8, 10, 39-40). This could be related to healthcare personnel unwilling to risk making a wrong referral (41-42). However, this can strengthen the parents' image that it's in the pediatric emergency department that they will receive the best care and create a search pattern that is inadequate (43). This can also cause contradictions between the different parts of the healthcare system (39). It should be added, however, that the majority of studies on telenursing show that telenurses generally advise seeking a lower level of care than the caller first intended (3, 38, 44).

The nurses in this study had perceptions about who and what information and advice to trust when caring for a febrile child. While stating that parents could find their own information, it was understood that this might be difficult for someone without healthcare education, and the nurses perceived themselves to be the experts. Earlier studies have shown that parents are just as aware as the nurses that it can be difficult to know what information is reliable; however, contrary to the perceptions of the nurses in this study, parents do seek, and get, help from both family and friends, as well as the internet, when they need information and advice (4, 32, 45-46). On the other hand, when information and advice came from healthcare personnel, it had the highest compliance (46), which may be a sign that information from healthcare personnel, as well as by parents is considered to be the most reliable information source.

The nurses in the present study described a balance between independence and having someone by one's side. Giving advice on fever is perceived as difficult, and the nurses sometimes need support from colleagues in their decision-making when advising parents on this matter, something that has been seen in previously studies on telenursing as well (37, 41). Advising via telephone is even more complicated, because the assessment is usually done through a parent. This type of work requires experience, which may be considered even more important than education (41).

Strengths and limitations

According to Lincoln and Guba (47), the trustworthiness of a qualitative study is founded on four pillars: *dependability, confirmability, credibility* and *transferability* (47). In order to ensure

For peer review only

dependability and *confirmability*, the authors have tried to provide a clear description on how the study was performed as well as citations to support the results. None of the authors have any type of relationship with the participants. The first author has experience advising parents when a child has a fever but has never worked in any of the participants' workplaces. Together with the research group, the first author continuously reflected on her own pre-understanding to avoid bias. Interviews were performed, since this may be considered the most common tool to collect data for this kind of study (23). To find all the different perceptions that can exist in a group, Larsson och Holmström (24) believes that approximately 20 interviews are sufficient (24). The 24 interviews that were conducted were considered an appropriate amount of data to handle and were judged to be rich in different perceptions. Due to the Covid-19 pandemic, the interviews were done via video-conference call, and although most of the participants were accustomed to this way of communicating, it could limit the participants' ways of expressing themselves. Investigator triangulation was used to ensure *credibility*. Two of the five authors had experience in the phenomenographic method, and all of the authors had experience in qualitative methods. A particularly large focus was on separating what belonged to first- and second-order perspectives, as this is a fundamental aspect of the phenomenographic analysis. The findings were continuously reflected on until consensus arose. To ensure that the reader can assess *transferability* to their own setting, settings and participants were described thoroughly. There may be differences in the educational and healthcare systems of different countries, though, which should be taken into consideration. Patients or the public were not involved in the design, or conduct, or reporting, or dissemination plans of this study.

Conclusion and relevance to clinical practice

Giving advice to parents when a child has a fever is a process where the nurse needs to listen, assess and give advice dependent on the situation. This is perceived as a balancing act where the nurse must make a correct assessment that depends on the parents' story. Creating a trusting relationship is perceived as necessary for parents to assimilate the advice that is provided. Surprisingly, what dominates are the nurses' perceptions of the inner qualities required to achieve a balance in the process. This shows the importance of experience and support from colleagues. This study can be used as a foundation for discussion and reflection, both for those who are about to learn this part of the nursing profession and for those who have experience. Responding to parents in a professional and empathetic manner and trying to achieve consensus provides better possibilities for parents to feel trust and for advice to be well received. It is a step in creating reassured parents who are strengthened in their self-care capacity, which in the long run relieves the burden on the healthcare organization and provides better accessibility. Future research should focus on how interactions between nurses and parents affect the outcome of the advice-giving process from the view of both parents and nurses.

For peer review only - http://bmjopen.bmj.com/site/about/guidelines.xhtml

Funding statement

This work was supported by Region Kronoberg grant number 969335.

Competing interests

None

Author contributions

All authors designed the study and drafted the manuscript. EW conducted the interviews and analyzed the data. CE and ILG actively participated in data analysis. MSL and AS were involved during the analysis process.

References

1. Gren C, Pedersen MK, Hasselager AB, et al. How parents express their worry in calls to a medical helpline: a mixed methods study. BMC Prim Care. 2022;23:80. doi: 10.1186/s12875-022-01680-4.

2. Kaminsky E, Carlsson M, Höglund AT, et al. Paediatric health calls to Swedish telenurses: a descriptive study of content and outcome. J Telemed Telecare. 2010;16(8):454-7. doi: 10.1258/jtt.2010.100110.

3. Keatinge D, Rawlings K. Outcomes of a nurse-led telephone triage service in Australia. Int J Nurs Pract. 2005;11:5-12. doi: 10.1111/j.1440-172X.2005.00495.x.

4. de Bont EG, Lepot JM, Hendrix DA, et al. Workload and management of childhood fever at general practice out-of-hours care: an observational cohort study. BMJ Open. 2015;5:e007365. doi: 10.1136/bmjopen-2014-007365.

5. Nokoff N, Brunner AM, Linakis JG, et al. Presentation to either the pediatric emergency department or primary care clinic for acute illness: the caregivers' perspective. Pediatr Emerg Care. 2014;30(3):146-50. doi: 10.1097/PEC.0000000000000082.

6. Drent AM, Brousseau DC, Morrison AK. Health Information Preferences of Parents in a Pediatric Emergency Department. Clin Pediatr (Phila). 2018;57(5):519-27. doi: 10.1177/0009922817730346.

For peer review only

7. Ellbrant J, Åkeson J, Karlsland Åkeson, P. Pediatric emergency department management benefits from appropriate early redirection of nonurgent visits. Pediatr Emerg Care 2015;31(2):95-100. doi: 10.1097/PEC.0000000000000348.

8. Kubicek K, Liu D, Beaudin C, et al. A profile of nonurgent emergency department use in an urban pediatric hospital. Pediatr Emerg Care. 2012;28(10):977-84. doi.org/10.1097/pec.0b013e31826c9aab

9. Smith V, Mustafa M, Grafstein E, et al. Factors Influencing the Decision to Attend a Pediatric Emergency Department for Nonemergent Complaints. Pediatr Emerg Care 2015;31(9):640-4. doi: 10.1097/PEC.0000000000000392.

10. O'Cathain A, Connell J, Long J, et al. 'Clinically unnecessary' use of emergency and urgent care: A realist review of patients' decision making. Health Expect. 2020;23:19-40. doi: 10.1111/hex.12995.

11. Swedish Nursing Association. Competence description for registered nurses, in Swedish (Kompetensbeskrivning för sjuksköterskor) [online]. 2017. https://www.swenurse.se/publikationer/kompetensbeskrivning-for-legitimerad-sjukskoterska (accessed 7 February 2023).

12. Halldórsdóttir S. (1996). Caring and uncaring encounters in nursing and health care. Developing a theory [dissertation]. Linköping; Linköping University; 1996. Medical Dissertation, No 493.

13. Monsma J, Richerson J, Sloand E. Empowering parents for evidence-based fever management: An integrative review. J Am Assoc Nurse Pract. 2015;27(4):222–9. doi: 10.1002/2327-6924.12152

14. Ellbrant JA, Åkeson J, Karlsland Åkeson P. Influence of awareness and availability of medical alternatives on parents seeking paediatric emergency care. Scand J Public Health. 2018;46(4):456-62. doi: 10.1177/1403494817735222

15. Swedish Healthcare Direct. When you call 1177, in Swedish (När du ringer 1177) [online]. 2023. http://www.1177.se/Om-1177/1177-sjukvardsradgivning/ (accessed 12 January 2023).

16. Anell A. The function, organization and finances of primary care - a literature review: Report to the inquiry A national coordinator for more efficient resource utilization in health care, in Swedish (Primärvårdens funktion, organisation och ekonomi – en litteraturöversikt: Rapport till utredningen En nationell samordnare för effektivare

For peer review only

resursutnyttjande inom hälso- och sjukvården). (S 2013:4). Socialdepartementet, 2015. 55 p. (Statens offentliga utredningar).

17. National Board of Health and Welfare. The mission of primary care. A survey of how the county council's mandate for primary care is formulated, in Swedish (Primärvårdens uppdrag. En kartläggning av hur landstingens uppdrag till primärvården är formulerade) [online]. 2016. https://www.socialstyrelsen.se/globalassets/sharepoint-dokument/artikelkatalog/ovrigt/2016-3-2.pdf (accessed 7 February 2023).

18. National Board of Health and Welfare. Guidance for child health care, in Swedish (Vägledning för barnhälsovården) [online]. 2014. https://www.socialstyrelsen.se/globalassets/sharepoint-dokument/artikelkatalog/vagledning/2014-4-5.pdf (accessed 7 February 2023).

19. Thompson AP, Nesari M, Hartling L, et al. Parents' experiences and information needs related to childhood fever: A systematic review. Patient Educ Couns. 2020;103(4):750-63. doi: 10.1016/j.pec.2019.10.004

20. Westin, E, Sund Levander, M. Parent's Experiences of Their Children Suffering Febrile Seizures. J Pediatr Nurs. 2018;38:68-73. doi: 10.1016/j.pedn.2017.11.001.

21. The delegation for increased accessibility in healthcare.The path to increased accessibility – long-term, strategic and in collaboration: interim report, in Swedish (Vägen till ökad tillgänglighet – långsiktig, strategisk och i samverkan: delbetänkande). (SOU 2021:59). [online]. https://www.regeringen.se/contentassets/b4d9ad7b41624ea79e9ff4be8d2cbf80/sou-2021_59.pdf (accessed 12 February 2023).

22. Marton F. Phenomenography – describing conceptions of the world around us. Instructional Science, 1981; 10(2):177-200. doi.org/10.1007/BF00132516

23. Larsson S. Qualitative Analysis The example of Phenomenography, in Swedish (Kvalitativ analys exemplet fenomenografi). Lund: Studentlitteratur; 1981

24. Larsson J, Holmström I. Phenomenographic or phenomenological analysis: does it matter? Examples from a study on anaesthesiologists' work. Int J Qual Stud Health Well-being. 2009;2:55-64. doi:10.1080/17482620601068105

25. World Medical Association. WMA Declaration of Helsinki - Ethical principles for medical research involving human subjects. [online]. 2022. https://www.wma.net/policies-post/wma-declaration-of-helsinki-ethical-principles-for-medical-research-involving-human-subjects/ (accessed 12 February 2023).

26. Greenberg ME. A comprehensive model of the process of telephone nursing. Journal of advanced nursing. 2009;65(12):2621-29. doi: 10.1111/j.1365-2648.2009.05132.x.

For peer review only

27. Kai J. Parents' difficulties and information needs in coping with acute illness in preschool children: a qualitative study. BMJ. 1996;313(7063):987-90. doi: 10.1136/bmj.313.7063.987.

28. Gustafsson S, Sävenstedt S, Martinsson J, et al. Need for reassurance in self-care of minor illnesses. J Clin Nurs. 2018;27(5-6):1183-91. doi: 10.1111/jocn.14157.

29. Kuijpers DL, Peeters D, Boom NC, et al. Parental assessment of disease severity in febrile children under 5 years of age: a qualitative study. BMJ Open. 2021;11:e042609. doi: 10.1136/bmjopen-2020-042609.

30. Lass M, Rahr Tatari C, Hoffmann Merrild C, et al. Contact to the out-of-hours service among Danish parents of small children - a qualitative interview study. Scand J Prim Health Care. 2018;36(2):216-23. doi: 10.1080/02813432.2018.1459431.

31. Peetoom KKB, Ploum LJL, Smits JJM, et al. Childhood fever in well-child clinics: a focus group study among doctors and nurses. BMC Health Serv Res 2016;16:240. doi: 10.1186/s12913-016-1488-1.

32. Thompson AP, Le A, Hartling L, et al. Fading Confidence: A Qualitative Exploration of Parents' Experiences Caring for a Febrile Child. J Clin Nurs. 2020;29(5-6):964-73. doi: 10.1111/jocn.15165.

33. Green JM, Spiby H, Hucknall C, et al. Converting policy into care: women's satisfaction with the early labour telephone component of the All Wales Clinical Pathway for Normal Labour. J Adv Nurs. 2012;68(10):2218-28. doi: 10.1111/j.1365-2648.2011.05906.x.

34. Gamst-Jensen H, Frischknecht Christensen E, Lippert F, et al. Self-rated worry is associated with hospital admission in out-of-hours telephone triage - a prospective cohort study. Scand J Trauma Resusc Emerg Med. 2020;28:53. doi: 10.1186/s13049-020-00743-8.

35. Cabral C, Ingram J, Hay AD, et al. "They just say everything's a virus"—Parent's judgment of the credibility of clinician communication in primary care consultations for respiratory tract infections in children: A qualitative study. Patient Educ Couns. 2014;95(2):248-53. doi: 10.1016/j.pec.2014.01.010.

36. Halls A, Van't Hoff C, Little P, et al. Qualitative interview study of parents' perspectives, concerns and experiences of the management of lower respiratory tract infections in children in primary care. BMJ Open. 2017;7:e015701. doi: 10.1136/bmjopen-2016-015701.

37. Eriksson I, Wilhsson M, Blom T, et al. Telephone nurses' strategies for managing difficult calls: A qualitative content analysis. Nurs Open. 2020;7(6):1671-79. doi: 10.1002/nop2.549.

For peer review only

38. Marklund B, Ström M, Månsson J, et al. Computer-supported telephone nurse triage: an evaluation of medical quality and costs. J Nurs Manag. 2007;15(2):180-7. doi: 10.1111/j.1365-2834.2007.00659.x.

39. Ernesäter A, Engström M, Holmström I, et al. Incident reporting in nurse-led national telephone triage in Sweden: the reported errors reveal a pattern that needs to be broken. J Telemed Telecare. 2010;16(5):243-7. doi: 10.1258/jtt.2009.090813.

40. McKenna G, Rogers A, Walker S, et al. The influence of personal communities in understanding avoidable emergency department attendance: qualitative study. BMC Health Serv Res. 2020;20:887. doi: 10.1186/s12913-020-05705-5.

41. Berntsson K, Eliasson M, Beckman L. Patient safety when receiving telephone advice in primary care - a Swedish qualitative interview study. BMC Nurs. 2022;21:24. doi: 10.1186/s12912-021-00796-9.

42. Brousseau DC, Nimmer MR, Yunk NL, et al. Nonurgent emergency-department care: analysis of parent and primary physician perspectives. Pediatrics 2011;127(2):e375-81. doi: 10.1542/peds.2010-1723.

43. Hiller MG, Caffery MS, Bégué RE. A Survey About Fever Knowledge, Attitudes, and Practices Among Parents. Clin Pediatr (Phila). 2019;58(6):677-80. doi: 10.1177/0009922819834276.

44. Sundberg A, Wahlberg AC, Zethraeus N, et al. Observational study of the implementation of telephone advice nursing in Sweden: did callers follow recommendations and did the rate of healthcare visits change? BMJ Open. 2021 Aug;11:e051233. doi: 10.1136/bmjopen-2021-051233.

45. Hamideh Kerdar S, Himbert C, Martin DD, et al. Cross-sectional study of parental knowledge, behaviour and anxiety in management of paediatric fever among German parents. BMJ Open. 2021;11:e054742. doi: 10.1136/bmjopen-2021-054742.

46. Gustafsson S, Vikman I, Axelsson K, et al. Self-care for minor illness. Prim Health Care Res Dev. 2015;16:71-8. doi: 10.1017/S1463423613000522.

47. Lincoln YS, Guba EG. Naturalistic inquiry. (1985). Newbury Park, CA: SAGE Publications.

For peer review only

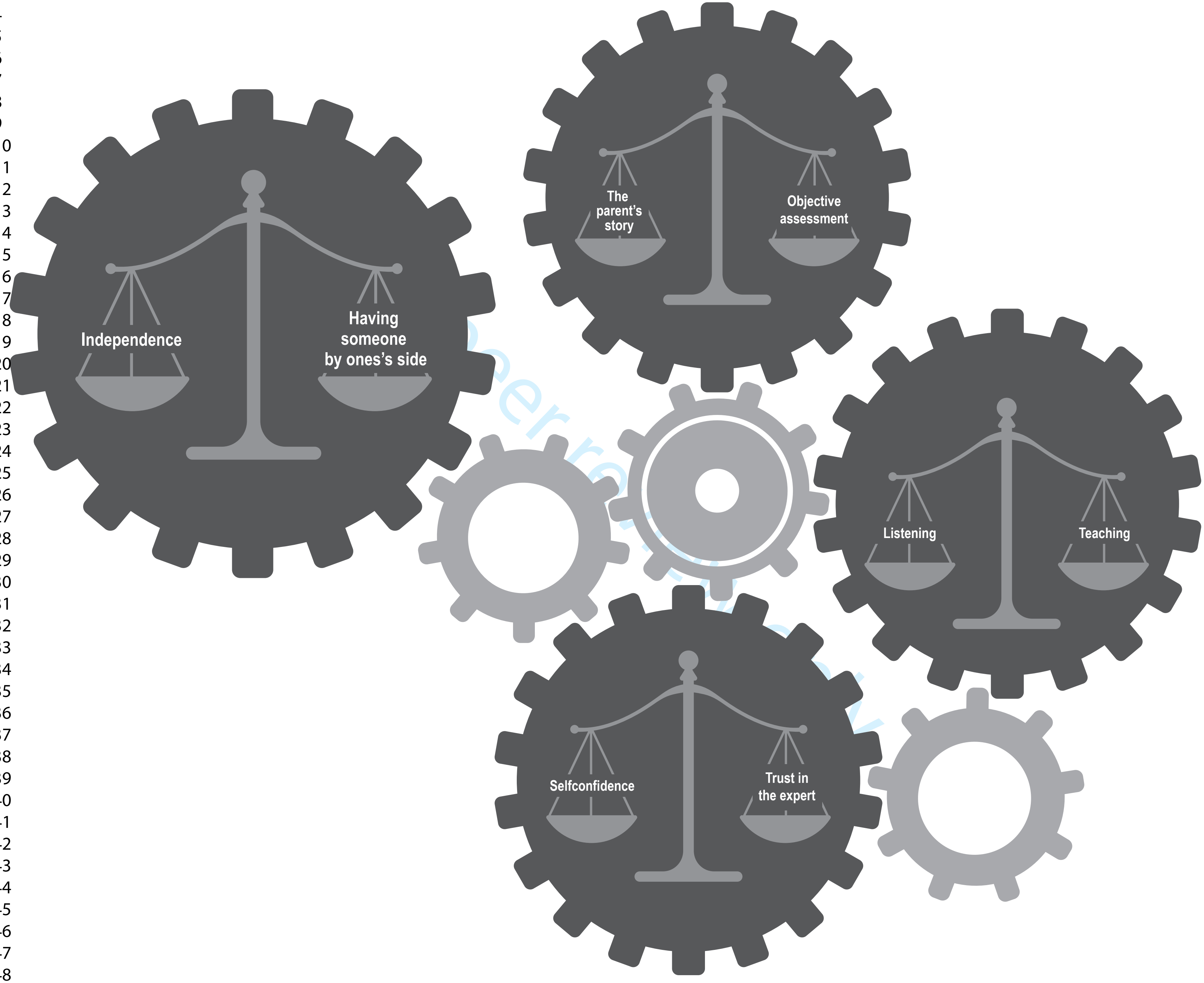
The parent's story
Objective assessment
Independence
Having someone by ones's side
Listening
Teaching
Selfconfidence
Trust in the expert

Standards for Reporting Qualitative Research (SRQR)*

http://www.equator-network.org/reporting-guidelines/srqr/

Page/line no(s).

Title and abstract

**Title** - Concise description of the nature and topic of the study Identifying the study as qualitative or indicating the approach (e.g., ethnography, grounded theory) or data collection methods (e.g., interview, focus group) is recommended	1/1-2
**Abstract** - Summary of key elements of the study using the abstract format of the intended publication; typically includes background, purpose, methods, results, and conclusions	1/4-24

Introduction

**Problem formulation** - Description and significance of the problem/phenomenon studied; review of relevant theory and empirical work; problem statement	2/1-36
**Purpose or research question** - Purpose of the study and specific objectives or questions	2/34-36

Methods

**Qualitative approach and research paradigm** - Qualitative approach (e.g., ethnography, grounded theory, case study, phenomenology, narrative research) and guiding theory if appropriate; identifying the research paradigm (e.g., postpositivist, constructivist/ interpretivist) is also recommended; rationale**	3/ 2-8
**Researcher characteristics and reflexivity** - Researchers' characteristics that may influence the research, including personal attributes, qualifications/experience, relationship with participants, assumptions, and/or presuppositions; potential or actual interaction between researchers' characteristics and the research questions, approach, methods, results, and/or transferability	11/ 2-6, 13-15
**Context** - Setting/site and salient contextual factors; rationale**	1/ 14-28<br>3/ 11-12<br>Table 1
**Sampling strategy** - How and why research participants, documents, or events were selected; criteria for deciding when no further sampling was necessary (e.g., sampling saturation); rationale**	3/ 11-20<br>11/ 7-10
**Ethical issues pertaining to human subjects** - Documentation of approval by an appropriate ethics review board and participant consent, or explanation for lack thereof; other confidentiality and data security issues	3/ 16-18<br>5/ 4-7

For peer review only - http://bmjopen.bmj.com/site/about/guidelines.xhtml

**Data collection methods** - Types of data collected; details of data collection procedures including (as appropriate) start and stop dates of data collection and analysis, iterative process, triangulation of sources/methods, and modification of procedures in response to evolving study findings; rationale**	4/ 2-3
**Data collection instruments and technologies** - Description of instruments (e.g., interview guides, questionnaires) and devices (e.g., audio recorders) used for data collection; if/how the instrument(s) changed over the course of the study	4/ 3-9
**Units of study** - Number and relevant characteristics of participants, documents, or events included in the study; level of participation (could be reported in results)	3/ 18-23<br>Table 1
**Data processing** - Methods for processing data prior to and during analysis, including transcription, data entry, data management and security, verification of data integrity, data coding, and anonymization/de-identification of excerpts	4/ 9-10
**Data analysis** - Process by which inferences, themes, etc., were identified and developed, including the researchers involved in data analysis; usually references a specific paradigm or approach; rationale**	4/ 11-16<br>Box 1<br>Table 2
**Techniques to enhance trustworthiness** - Techniques to enhance trustworthiness and credibility of data analysis (e.g., member checking, audit trail, triangulation); rationale**	10/ 42-43<br>11/ 1-22

Results/findings

**Synthesis and interpretation** - Main findings (e.g., interpretations, inferences, and themes); might include development of a theory or model, or integration with prior research or theory	5/9-15<br>6/1-6, 11-24, 28-34<br>7/1-14, 19-31, 36-39<br>8/1-3, 7-13, 15-23, 29-38<br>Figure 1
**Links to empirical data** - Evidence (e.g., quotes, field notes, text excerpts, photographs) to substantiate analytic findings	6/ 7-10, 25-26, 35-39<br>7/ 15-17, 32-25<br>8/ 4-6, 24-28<br>9/ 1-3

Discussion

**Integration with prior work, implications, transferability, and contribution(s) to the field -** Short summary of main findings; explanation of how findings and conclusions connect to, support, elaborate on, or challenge conclusions of earlier scholarship; discussion of scope of application/generalizability; identification of unique contribution(s) to scholarship in a discipline or field	9/5-41<br>10/1-39
**Limitations** - Trustworthiness and limitations of findings	10/ 42-43<br>11/ 1-22

Other

For peer review only - http://bmjopen.bmj.com/site/about/guidelines.xhtml

**Conflicts of interest** - Potential sources of influence or perceived influence on study conduct and conclusions; how these were managed	12/ 4-5
**Funding** - Sources of funding and other support; role of funders in data collection, interpretation, and reporting	12/ 1-2

*The authors created the SRQR by searching the literature to identify guidelines, reporting standards, and critical appraisal criteria for qualitative research; reviewing the reference lists of retrieved sources; and contacting experts to gain feedback. The SRQR aims to improve the transparency of all aspects of qualitative research by providing clear standards for reporting qualitative research.

**The rationale should briefly discuss the justification for choosing that theory, approach, method, or technique rather than other options available, the assumptions and limitations implicit in those choices, and how those choices influence study conclusions and transferability. As appropriate, the rationale for several items might be discussed together.

Reference:
O'Brien BC, Harris IB, Beckman TJ, Reed DA, Cook DA. **Standards for reporting qualitative research: a synthesis of recommendations.** *Academic Medicine*, Vol. 89, No. 9 / Sept 2014 DOI: 10.1097/ACM.0000000000000388

For peer review only

Advising parents when their child has a fever – a phenomenographic analysis of nurses' perceptions when working at a telephone helpline, at primary care or at a children's emergency department

Journal:	*BMJ Open*
Manuscript ID	bmjopen-2023-074823.R1
Article Type:	Original research
Date Submitted by the Author:	11-Dec-2023
Complete List of Authors:	Westin, Emma; Linnaeus University - Vaxjo Campus, Department of Health and Caring Sciences ; Region Kronoberg, Department of pediatrics<br>Gustafsson, Ingrid; University College of Boras Faculty of Caring Science Work Life and Social Welfare, Department of caring sciences; Linnaeus University Faculty of Health and Life Sciences, Department of health and caring sciences<br>Svensson, Anders; Linnaeus University Faculty of Health and Life Sciences, Health and Caring Science; Region Kronoberg, Department of Ambulance Service<br>Sund-Levander, Märta; Linkopings universitet, Department of Health and Care<br>Elmqvist, Carina; Linnaeus University Faculty of Health and Life Sciences, Dept. of Health and Caring Sciences, Linnaeus University; Region Kronoberg, Head of Research
**Primary Subject Heading**:	Nursing
Secondary Subject Heading:	Paediatrics, Qualitative research
Keywords:	Nursing Care, PAEDIATRICS, QUALITATIVE RESEARCH

I, the Submitting Author has the right to grant and does grant on behalf of all authors of the Work (as defined in the below author licence), an exclusive licence and/or a non-exclusive licence for contributions from authors who are: i) UK Crown employees; ii) where BMJ has agreed a CC-BY licence shall apply, and/or iii) in accordance with the terms applicable for US Federal Government officers or employees acting as part of their official duties; on a worldwide, perpetual, irrevocable, royalty-free basis to BMJ Publishing Group Ltd ("BMJ") its licensees and where the relevant Journal is co-owned by BMJ to the co-owners of the Journal, to publish the Work in this journal and any other BMJ products and to exploit all rights, as set out in our licence.

The Submitting Author accepts and understands that any supply made under these terms is made by BMJ to the Submitting Author unless you are acting as an employee on behalf of your employer or a postgraduate student of an affiliated institution which is paying any applicable article publishing charge ("APC") for Open Access articles. Where the Submitting Author wishes to make the Work available on an Open Access basis (and intends to pay the relevant APC), the terms of reuse of such Open Access shall be governed by a Creative Commons licence – details of these licences and which Creative Commons licence will apply to this Work are set out in our licence referred to above.

Other than as permitted in any relevant BMJ Author's Self Archiving Policies, I confirm this Work has not been accepted for publication elsewhere, is not being considered for publication elsewhere and does not duplicate material already published. I confirm all authors consent to publication of this Work and authorise the granting of this licence.

For peer review only

Advising parents when their child has a fever – a phenomenographic analysis of nurses' perceptions when working at a telephone helpline, at primary care or at a children's emergency department

For peer

Abstract

Objectives: To describe nurses' perceptions of advising parents when their child has a fever.

Design/method: Inductive, descriptive study with a qualitative, phenomenographic approach.

Participants and setting: A purposive sampling was used. To be included, the 24 online interviewed nurses had to have experience advising parents of febrile children between birth and five years of age. They were recruited from three different parts of the healthcare system from four regions in the south of Sweden.

Results: The nurses described advising parents when their child has a fever as four different kinds of balancing acts: Balancing between the parents' story and objective assessment, balancing between listening and teaching, balancing between self-confidence and trust in the expert, and balancing between independence and having someone by one's side.

Conclusions: Giving advice to parents when a child has a fever is a process where the nurse needs to listen, assess and give advice based on the situation. This requires a correct assessment that depends on the parents' story. Creating a trusting relationship is perceived as necessary for parents to assimilate the advice that is provided. What dominates are the nurses' perceptions of the inner qualities required to achieve a balance in the process, for example the importance of experience and security in their professional role, while it is also necessary to get support from colleagues.

Strengths and limitations of this study

- Two of the five authors have experience in the phenomenographic method, and all of the authors have experience in qualitative methods.
- The first author carried out and transcribed all the interviews.
- There is great variation in the participants workplace, work experience and age.

- The interviews were done via video-conference call, and although most of the participants were accustomed to that way of communicating, this could limit the participants' ways of expressing themselves.

Introduction

In Sweden, parents can generally seek advice via telephone nursing services or within primary care (1). Advising is defined as helping the parents assess the need for healthcare, give advice and information about measures that the parents can do themselves, and, if necessary, refer to appropriate level of care (2). The telephone helpline 1177 Vårdguiden (henceforth 1177) are accessible around the clock all year round. The main focus for the telenurses at 1177 is to advise on different health problems (3). To support their advice, they use a computerized decision support tool (CDST); the same system is also frequently used by nurses who work in primary care. Primary care centers are responsible for preventing, diagnosing and treating illnesses, and, if necessary, sending referrals to specialist care (4). They must offer services for both planned and unplanned visits in general medicine during the day, as well as some on-call services. Primary care also includes child healthcare (5). The goal of child healthcare is to promote health and prevent illness in children from birth until the child starts school by following the child's development and health status and providing support to parents (6). If the child has acute symptoms, the parents should go directly to the pediatric emergency department, whose main purpose is to provide fast and highly specialized care for acute, sometimes life-threatening conditions. However, it is common for parents to go there for minor illnesses; in those cases, advice from a nurse may sometimes be the only course of action (7).

Feverish children cause concerns and are among the most common reasons why parents contact telephone nursing services (8-10), primary care (11-12) or children's emergency departments (7, 12-15). The most common expectations parents have is to get their child a physical examination by a physician, and to receive reassurance and information about warning signs (16). The initial meeting within the healthcare system is usually with a nurse, who is responsible both for creating a trusting relationship and for providing adequate information (17). According to Halldórsdóttir (18), this first encounter is decisive in whether the meeting and the relationship will be perceived as caring or non-caring (18). It is important that the nurses can identify the parents' needs, which can vary depending on background, expectations and previous experience, in order to decide what strategies that can be used to empower them (19). Previous studies have examined parents' views of having a child with fever, with or without contact with the healthcare system (20, 21). It is also recognized that nurses can play an important role in providing knowledge and advice to these parents (19). However, previous research shows that advising can be experienced as an advanced and demanding task (22). Knowledge about nurses' perceptions is needed in order to prepare and support nurses taking on this role. This study therefore aims to describe nurses' perceptions of advising parents when their child has a fever, in a Swedish context.

For peer review only

Methods

Design

inductive, descriptive study with a qualitative approach was conducted i.e., phenomenography which describes the different perceptions that exist of a phenomenon. This is referred to by Marton (23) as the second-order perspective. The first-order perspective concerns objective observations, i.e., facts (23). Perceptions, on the other hand, are unspoken, implied and unreflected. It is perceptions that form the frame of reference for our thinking and the basis for our opinions (24).

Participants

Purposive sampling was used to obtain a rich and varied understanding of the phenomenon. That included nurses from four different regions in southern Sweden from three different parts of the healthcare system; the telephone helpline 1177 Vårdguiden; primary healthcare, including child healthcare; and a pediatric emergency department. To be included, the nurses had to have experience advising parents of febrile children between birth and five years of age. The first author contacted the department head at each workplace, who then sent out information about the study to the nurses. Those who then chose to participate contacted the author directly. The participants received both written and oral information about the study and provided informed written consent. They were informed that participation was voluntary and that they had the right to cancel their participation in the study at any time. To find all the different perceptions that can exist in a group, Larsson och Holmström (25) believes that approximately 20 interviews are sufficient, therefor the goal was to get between 6-8 participants from each part of the healthcare system and with a geographical spread. This resulted in a total of 24 participants. The participants varied between 27 and 64 years (average 45), and their experience varied from 11 months to 25 years. Of the 24 nurses, 13 had at least one specialist nurse education, most commonly pediatric nurse or primary healthcare nurse (see Table 1).

Table 1. Demographics of the 24 nurses.

	Telephone helpline 1177 (n=8)	Primary health care central and child health central (n=8)	Pediatric emergency department (n=8)
**Gender (F/M)**	8/0	8/0	6/2

For peer review only - http://bmjopen.bmj.com/site/about/guidelines.xhtml

**Age (mean years)**	48-64 (55,75)	34-64 (46,38)	27-44 (34,25)
**Specialist nurse education (yes/no)**	2/6	7/1	4/4
**Work experience (mean years)**	0,9-15 (5.6)	3,5-25 (8.6)	2,5-10 (7.8)

Data collection

The interviews were conducted via Zoom by the first author during the period from March 2021 to October 2021. Data were collected through semi-structured interviews. The interviews all began with the same open-ended question: "What is advising parents whose child has a fever for you?” Followed by a number of predefined questions according to an interview guide (see supplementary file 1). Follow-up questions such as "Can you tell me more?" and “What do you mean when you say…?” were used in order for the participants to reflect on the phenomenon and deepen their reasoning. Two pilot interviews were conducted, and since they did not lead to any changes in the interview guide, both were included in the study. The interviews lasted between 22 and 51 minutes (average 37 minutes) and were transcribed verbatim. Only the person who conducted the interviews had access to the recorded interviews, i.e., the first author. While the interviews were transcribed, they were de-identified from anything that could reveal the participant's identity. Only those who participated in the analysis had access to the transcriptions.

Data analysis

Data analysis was performed following the seven-steps model of Larsson and Holmström (25) (see Box 1). The analysis is not linear, but rather a back and forth motion between the whole and the parts. The analysis was mainly performed by the first, second and last author, but all authors participated and discussed the various steps during the process. The first, second and last authors started out each step in the analysis together, then the first author finished each step but the second and last authors reflected on and verified the outcome. Regularly scheduled meetings were done with the whole group.

Box 1. The seven steps of data analysis, according to Larsson and Holmström (25).

1. The text of the transcribed interviews was read through repeatedly.

For peer review only

For peer review only - http://bmjopen.bmj.com/site/about/guidelines.xhtml

2. The answers to the interview-questions were marked. This second step was done again and again to make sure that the marked text correlated to the questions.
3. In the third step, the authors looked in the marked text for what the focus of the nurse's attention was and how she/he described her/his way of advising parents when their child has a fever. This resulted in a preliminary description of each nurse´s predominant way of understanding their work, in other words, their perceptions.
4. The descriptions was grouped into categories based on similarities and differences. This step was done and redone several times, like a journey between parts and whole to ensure that the categories were found in the material and vice versa.
5. Non-dominant ways of understanding were searched out. For an overview of dominant (++) and non-dominant (+) ways of understanding nurses' perceptions of advising parents when their child has a fever, see Table 2.
6. The hierarchical way the categories were related to one another were defined, structuring the outcome space.
7. Based of outcome space, each category of description was assigned a metaphor, in this case, each category came out as four different kinds of balancing acts.

For peer review only

Table 2. Overview of dominant (++) and non-dominant (+) ways of understanding nurses' perceptions of advising parents when their child has a fever.

Interview	The parents' story and objective assessment	Listening and teaching	Self-confidence and trust in the expert	Independence and having someone by one's side
1	+	+	++	++
2	+	++	++	++
3	+	+	+	++
4	++	+	++	++
5	++	++		+
6	++	++	++	++
7	+	++	+	++
8	++	++	+	++
9	+	++	++	++
10	+	+	+	++
11	+	+	+	+
12	++	+	+	++
13	+	+	+	++
14	+	++	++	++
15	++	+	+	++
16	+	+	++	++
17	++	++	+	++
18	+	+	+	++
19	+	+	+	++
20		+	+	++
21	+		+	+
22	+	+	+	++
23		+	+	+
24	+		+	++

Ethical considerations

Ethical approval was granted by the Swedish Ethical Review Authority (Dnr: 2020-03731). In accordance with the Declaration of Helsinki, the integrity and dignity of the participants have been respected in that their data and interviews were treated confidentially (26).

Patient and public involvement

Patients or the public were not involved in the design, conduct, reporting, or dissemination plans of this study.

Results

The nurses perceive advising parents when their child has a fever as four different kinds of balancing acts: Balancing between the parents' story and objective assessment, balancing between listening and teaching, balancing between self-confidence and trust in the expert, and balancing between independence and having someone by one's side. All these balancing acts are part of and influence the process of giving advice, but the balancing between independence and having someone by one´s side was more dominantly expressed than the others according to table 2. For a schematic view of the outcome space, see Figure 1.

Balancing between the parents' story and objective assessment

The nurses perceive advising on fever as a balance between the parents' story and an objective assessment. Not seeing the sick child, as with telenursing, or when the nurse and parent don't speak the same language, is perceived as an additional difficulty. The nurse's objective assessment is based on the parents' subjective interpretation of the child's symptoms and their ability to describe them.

"There, we are also dependent on the parents to actually be able to make an assessment, so they may be dependent on us to get answers, but I am just as dependent on their answers to be able to make my assessment // it's quite interesting actually that it's not just them who need us, but we actually need them, and they know their child best". Q17

The nurses perceive that they are dependent on the parents' stories, but the parents' worries may cause them to assess their children as sicker than they are; at the same time, parents know their children best and their concerns should be taken seriously. This interaction is as a collaboration between nurse and parent where the expertise of the parents concerning their children needs to be respected. The nurses perceive that parents

For peer review only

need to provide enough information about the wellbeing of their children for nurses to be able to make an assessment, which requires good communication skills. Worry is perceived as potentially blocking this knowledge and affect the ability of parents to accurately assess their child's fever. The nurses' perceptions are that the degree of worry can be influenced by several different factors such as number of children, personality, culture, resources and previous experiences. The nurse's perceptions are that some parents have experienced a serious event in connection with a fever; others have read scary stories that are shared in the media and on the internet. Parents from countries where serious infections with fever are more common were perceived by the nurses as having greater worry about fever.

"The difficult thing is that there is a lot, there can be a lot of worry and then you don't really get the facts, but it is the worries that comes out". J10

For peer review only

Balancing between listening and teaching

The nurses perceive advising about fever as a balance between listening and teaching. Providing information about fever is considered part of a nurse's job, and the interaction between parents and nurses plays a role in how advice is received. It's not just about teaching general knowledge in case of fever, which is something that is simple in and of itself, but the nurse has to listen to the needs of parents and start from there in order to address their specific concerns.

"It will be to create a trust and then to make a knowledge inventory and then come along, with the information, or the advice that I want to give. If I start by giving advice, I don't think the family sees themselves being seen, then it becomes more dismissive even though it is well-intentioned on my part that I want to help. So it has to happen in the right order there" H8

When time is constrained, advising may become more standardized and less based on individual needs. This is perceived as creating a feeling in the parents that they are not being taken seriously. The nurses also perceive that sometimes consensus regarding what is classified as a serious illness is lacking, but regardless of what their own assessment is, the parents' concerns need to be confirmed so they feel they are being heard. This includes, for example, booking an appointment to see a doctor even if the nurse assesses this as being unnecessary. At the same time, the nurses perceive that the needs and wishes of the parents must be weighed against the resources of the healthcare system. The nurses' perceptions are that worried parents have the right to seek medical care, but there are benefits to consensus when it comes to, for example, the waiting time at the children's emergency department. In the absence of consensus, the likelihood of parents following such advice is perceived as reduced. In these cases, it is considered likely that the parents will seek additional advice, either at the same place or elsewhere. Taking the parents seriously, confirming their concerns and explaining the basis for the assessment are perceived as tools for achieving consensus.

"It's important not to diminish their worries but to show that you listen to them and that it's okay that they're worried. But you have to explain to them, if it's the case that we're not worried, why not in that case so that they understand our work". E5

Balancing between self-confidence and trust in the expert

The nurses' perceptions are that advising on fever is a balance between self-confidence and trust in the expert. Nurses at the children's emergency department are perceived as having the most expertise advising parents, most often using their own knowledge and local directions as guidance. Nurses in other parts of the healthcare system often described using a computerized decision support tool, CDST. It is considered evidence-based support, mainly for nurses who lack experience, but it is also perceived as requiring some experience to be interpreted correctly. CDST provides a structure and has clear directives when it comes to fever. At the same time, it can be perceived as undifferentiated and limited in more complex cases, and in such cases some nurses place more trust in their own knowledge. The fact that some nurses use CDST and some don't may result in parents receiving different answers depending on where they seek advice. This is perceived as causing confusion and sometimes resulting in contradictions between nurses in different parts of the healthcare system.

"Those who have done this decision support, they are specialists, so it's not someone sitting at home and tinkering with this // then, as I said, you have to have your own experience with you, also as a nurse, a newbie, newly trained nurse would not have been so appropriate perhaps to put... in advising, it is not, it is not so simple, so it is not". N14

The nurses perceive though, that the parents generally trust their advice, even if stories about when care has failed to detect and treat serious illness can reduce trust. The trustworthiness is perceived to increase if several people (or sources) say the same thing, if the assessment involves a physician or if the nurse and the parent have an established relation. Establishing trust via phone poses a greater challenge than meeting someone in person. The nurses perceive themselves as providing the most reliable and evidence-based advice and that parents see them as experts and often trust them over themselves.

"There is no opinion in that [the advice], but that it is actually a science that we rely on, I think it is a support that we are actually nurses that they talk to, it's not just anyone, like a grandmother or their mother" T20

Parents are perceived as having different abilities with regard to seeking knowledge about their child's fever, but it's generally perceived as being difficult to sift through all the available information and assess what is reliable. The nurses perceive that this information overload makes it difficult for parents to fully trust their own knowledge or that of their relatives. The nurses also expressed that even if the parents have general knowledge of fever, they may need help to assess the child's condition and require confirmation that their knowledge is being applied correctly to the situation.

For peer review only

Balancing between independence and having someone by one's side

The nurses perceive that advising on fever is a balance between independence and having someone by one's side. Both training and experience are required, especially on the phone, because fever can represent both harmless and serious conditions. This is also perceived as involving great responsibility as it requires caring for parents as well as the child. The nurse's perceptions are that they need to feel secure in their professional role and not be distressed by the parents' worries. Independently assessing a child with a fever can be perceived as a challenge, especially if it involves a younger child. With increasing experience and knowledge comes independence, but it is still important to have access to support, to have someone by one's side.

"But I think I have the greatest support of my colleagues, so partly I think, despite having worked for a long time, I think it's quite nice that one of my colleagues, it could be the assistant nurse, has also seen the child and says the same as me, that I'm not worried. They have, after all, are often very experienced, have seen a lot of children. Then I feel even more confident in my assessment and can provide better support". B2

This is also perceived by the nurses as being true for the parents. Parents today, though, are perceived as being fearful of fever, of lacking knowledge and support from their relatives, and instead need to seek this support from the healthcare system. This is by the nurses perceived to be one of the most common reasons for parents to seek advice. Therefore, the nurses perceive that parents need and expect healthcare to be accessible. Providing a plan for how parents should deal with their children having a fever and where to turn when this occurs is perceived by the nurses as a way of increasing the independence of parents, by giving them tools to take control of their situation. This can be combined with a follow-up call, where the nurse can determine whether the parents followed the advice that was given and can evaluate the results. This is perceived by the nurses as them supporting the parents.

"Because then I show that I actually care if the situation has improved or not, if they get the help they need, that someone cares about them and that they are not standing there alone in case something happens". D4

Discussion

This study understands nurses' perceptions of advising parents when their child has a fever as four different but related balancing acts. Listening to the parents, making assessments and offering advice can be considered the foundation of the advice-giving process, but the nurses' perceptions show that personal characteristics and peer support are important parts of the process. Overall, these results share similarities with those of Greenberg (27), who has previously described telephone nursing, however as a process comprising three phases. Phase one includes listening to the caller's story and gathering information. In the second phase, the nurse assesses the problem and determines the proper interventions, sometimes with the

For peer review only

support of a colleague. The third phase is called output and consists of different nursing actions such as referring to primary care or offering advice for self-management, reassurance and validation. This also describes personal characteristics of the nurse, for example, experience, as influencing factors of the telenursing process (27).

The nurses in the present study perceive difficulties in balancing between the parents' stories and an objective assessment sometimes as a result of difficulties in communication. Earlier studies show that parents often assess and express symptoms differently than healthcare personnel. For example, parents often start from the view of a child's normal behavior and judge the health of the child in relation to how much the behavior deviates from the usual (28-30). Parents may find it difficult to assess and express specific symptoms such as dehydration and breathing problems if they have not experienced them before (30). Conversely, healthcare personnel usually assess illness based on specific symptoms, which can lead to mistakes in communication (31). Greenberg (27) emphasizes that nurses interpreting between healthcare information and information that the caller can understand serves as an important component that links together different aspects of the telenursing process (27). The nurses in the present study perceived that parents' worry affects the way parents assess the symptoms of their children. Previous studies show that it can be difficult for parents to assess their sick child and know when it is time to seek medical care or when self-care is enough (32-33). Gamst-Jensen et al (34) shows that in telephone triage there is a relationship between parents' self-rated anxiety and hospitalization. The perceptions of the nurses in this study suggest that it is important to take parents' concerns into account whether you talk to the parents on the phone or meet them in person.

The nurses in the present study perceive that there had to be a balance between listening and teaching and that their relationship with the parents would decide whether they could reach them with their advice. Halldórsdóttir (18) argues that in order to achieve a caring relationship, the nurse must appear to genuinely care. The nurse needs to build a bridge where the parent feels a sense of belonging in the meeting. If, on the other hand, the parent feels that the nurse is lacking in caring and doesn't want or can't meet, a wall is built that makes it difficult to communicate and create trust (18). For example, the nurses in the present study described a gatekeeping role; even though the nurses wanted to provide service, they felt limited by the resources of the healthcare system. Being refused access to the desired healthcare can give a feeling of not being taken seriously (29, 35), which can then build a wall between parents and nurses. If there is a disagreement about how serious the child's illness is and the nurse fails to explain her assessment, it can lead to mistrust of the given advice. If the parents are not satisfied with the advice, there is a risk that they will seek advice again in the near future (36-37). To some extent, this may be due to the parents failing to regain a sense of control over the situation (28, 35).

The nurses in the present study expressed a perceived hierarchy within the healthcare system with regard to who was considered to be experts. A common reason for parents turning to the pediatric emergency department for a minor illness is that they have a higher level of trust in specialist care (often in combination with poor availability in primary care) (14-16, 22, 38). However, it has also been shown that within the healthcare system there is a large proportion of incorrect referrals to the pediatric emergency department (14, 16, 39-40). This could be

For peer review only

related to healthcare personnel unwilling to risk making a wrong referral (41-42). This can strengthen the parents' image that it's in the pediatric emergency department that they will receive the best care and create a search pattern that is inadequate (43). This can also cause contradictions between the different parts of the healthcare system (39). It should be added, however, that the majority of studies on telenursing show that telenurses generally advise seeking a lower level of care than the caller first intended (10, 38, 44).

The nurses in this study had perceptions about who and what information and advice to trust when caring for a febrile child. While stating that parents could find their own information, it was understood that this might be difficult for someone without healthcare education, and the nurses perceived themselves to be the experts. Earlier studies have shown that parents are just as aware as the nurses that it can be difficult to know what information is reliable; however, contrary to the perceptions of the nurses in this study, parents do seek, and get, help from both family and friends, as well as the internet, when they need information and advice (11, 33, 45-46). On the other hand, when information and advice came from healthcare personnel, it had the highest compliance (46), which may be a sign that information from healthcare personnel, as well as by parents is considered to be the most reliable information source.

The nurses in the present study perceive a balance between independence and having someone by one's side. Giving advice on fever is perceived as difficult, and the nurses sometimes need support from colleagues in their decision-making when advising parents on this matter, something that has been seen in previously studies as well, however, these studies have focused solely on telenursing (22, 41). Advising via telephone is even more complicated, because the assessment is usually done through a parent. This type of work requires experience, which may be considered even more important than education (41).

Strengths and limitations

According to Lincoln and Guba (47), the trustworthiness of a qualitative study is founded on four pillars: *dependability, confirmability, credibility* and *transferability* (47). In order to ensure *dependability* and *confirmability*, the authors have tried to provide a clear description on how the study was performed as well as citations to support the results. None of the authors have any type of relationship with the participants. The first author has experience advising parents when a child has a fever but has never worked in any of the participants' workplaces. Together with the research group, the first author continuously reflected on her own pre-understanding to avoid bias. Interviews were performed, since this may be considered the most common tool to collect data for this kind of study (24). Since Larsson och Holmström (25) believes that approximately 20 interviews are sufficient to find all the different perceptions that can exist in a group, the 24 interviews that were conducted were considered an appropriate amount of data to handle and they were judged to be rich in different perceptions. Due to the Covid-19 pandemic, the interviews were done via video-conference call, and although most of the participants were accustomed to this way of communicating, it could limit the participants' ways of expressing themselves. Investigator triangulation was used to ensure *credibility*. Two of the five authors had experience in the phenomenographic method, and all of the authors had experience in qualitative methods. A particularly large focus was on separating what

For peer review only

belonged to first- and second-order perspectives, as this is a fundamental aspect of the phenomenographic analysis. The findings were continuously reflected on until consensus arose. To ensure that the reader can assess *transferability* to their own setting, settings and participants were described thoroughly. There may be differences in the educational and healthcare systems of different countries, though, which should be taken into consideration.

Conclusion and relevance to clinical practice

Giving advice to parents when a child has a fever is a process where the nurse needs to listen, assess and give advice based on the situation. This requires a correct assessment that depends on the parents' story. Creating a trusting relationship is perceived as necessary for parents to assimilate the advice that is provided. What dominates are the nurses' perceptions of the inner qualities required to achieve a balance in the process, for example the importance of experience and security in their professional role, while it is also necessary to get support from colleagues. This study can be used as a foundation for discussion and reflection for nurses who work with this type of advising, but also as teaching material for students. It can also help nurses from different parts of the healthcare system to understand each other's work. Future research should focus on how interactions between nurses and parents affect the outcome of the advice-giving process from the view of both parents and nurses.

Funding statement

This work was supported by Region Kronoberg grant number 969335.

Competing interests

None

Author contributions

All authors (EW, IG, AS, MSL and CE) designed and planned the study together. EW was responsible for the data collection i.e. conducted and transcribed the interviews. The analysis was mainly performed by EW, IG and CE, but all authors participated and discussed the various steps during the process. All authors drafted, edited and finally approved the manuscript.

References

1. Ellbrant JA, Åkeson J, Karlsland Åkeson P. Influence of awareness and availability of medical alternatives on parents seeking paediatric emergency care. Scand J Public Health. 2018;46(4):456-62. doi: 10.1177/1403494817735222

For peer review only

2. The delegation for increased accessibility in healthcare. The path to increased accessibility – long-term, strategic and in collaboration: interim report, in Swedish (Vägen till ökad tillgänglighet – långsiktig, strategisk och i samverkan: delbetänkande). (SOU 2021:59). [online]. https://www.regeringen.se/contentassets/b4d9ad7b41624ea79e9ff4be8d2cbf80/sou-2021_59.pdf (accessed 12 February 2023).

3. Swedish Healthcare Direct. When you call 1177, in Swedish (När du ringer 1177) [online]. 2023. http://www.1177.se/Om-1177/1177-sjukvardsradgivning/ (accessed 12 January 2023).

4. Anell A. The function, organization and finances of primary care - a literature review: Report to the inquiry A national coordinator for more efficient resource utilization in health care, in Swedish (Primärvårdens funktion, organisation och ekonomi – en litteraturöversikt: Rapport till utredningen En nationell samordnare för effektivare resursutnyttjande inom hälso- och sjukvården). (S 2013:4). Socialdepartementet, 2015. 55 p. (Statens offentliga utredningar).

5. National Board of Health and Welfare. The mission of primary care. A survey of how the county council's mandate for primary care is formulated, in Swedish (Primärvårdens uppdrag. En kartläggning av hur landstingens uppdrag till primärvården är formulerade) [online]. 2016. https://www.socialstyrelsen.se/globalassets/sharepoint-dokument/artikelkatalog/ovrigt/2016-3-2.pdf (accessed 7 February 2023).

6. National Board of Health and Welfare. Guidance for child health care, in Swedish (Vägledning för barnhälsovården) [online]. 2014. https://www.socialstyrelsen.se/globalassets/sharepoint-dokument/artikelkatalog/vagledning/2014-4-5.pdf (accessed 7 February 2023).

7. Ellbrant J, Åkeson J, Karlsland Åkeson, P. Pediatric emergency department management benefits from appropriate early redirection of nonurgent visits. Pediatr Emerg Care 2015;31(2):95-100. doi: 10.1097/PEC.0000000000000348.

8. Gren C, Pedersen MK, Hasselager AB, et al. How parents express their worry in calls to a medical helpline: a mixed methods study. BMC Prim Care. 2022;23:80. doi: 10.1186/s12875-022-01680-4.

9. Kaminsky E, Carlsson M, Höglund AT, et al. Paediatric health calls to Swedish telenurses: a descriptive study of content and outcome. J Telemed Telecare. 2010;16(8):454-7. doi: 10.1258/jtt.2010.100110.

10. Keatinge D, Rawlings K. Outcomes of a nurse-led telephone triage service in Australia. Int J Nurs Pract. 2005;11:5-12. doi: 10.1111/j.1440-172X.2005.00495.x.

For peer review only

11. de Bont EG, Lepot JM, Hendrix DA, et al. Workload and management of childhood fever at general practice out-of-hours care: an observational cohort study. BMJ Open. 2015;5:e007365. doi: 10.1136/bmjopen-2014-007365.

12. Nokoff N, Brunner AM, Linakis JG, et al. Presentation to either the pediatric emergency department or primary care clinic for acute illness: the caregivers' perspective. Pediatr Emerg Care. 2014;30(3):146-50. doi: 10.1097/PEC.0000000000000082.

13. Drent AM, Brousseau DC, Morrison AK. Health Information Preferences of Parents in a Pediatric Emergency Department. Clin Pediatr (Phila). 2018;57(5):519-27. doi: 10.1177/0009922817730346.

14. Kubicek K, Liu D, Beaudin C, et al. A profile of nonurgent emergency department use in an urban pediatric hospital. Pediatr Emerg Care. 2012;28(10):977-84. doi.org/10.1097/pec.0b013e31826c9aab

15. Smith V, Mustafa M, Grafstein E, et al. Factors Influencing the Decision to Attend a Pediatric Emergency Department for Nonemergent Complaints. Pediatr Emerg Care 2015;31(9):640-4. doi: 10.1097/PEC.0000000000000392.

16. O'Cathain A, Connell J, Long J, et al. 'Clinically unnecessary' use of emergency and urgent care: A realist review of patients' decision making. Health Expect. 2020;23:19-40. doi: 10.1111/hex.12995.

17. Swedish Nursing Association. Competence description for registered nurses, in Swedish (Kompetensbeskrivning för sjuksköterskor) [online]. 2017. https://www.swenurse.se/publikationer/kompetensbeskrivning-for-legitimerad-sjukskoterska (accessed 7 February 2023).

18. Halldórsdóttir S. (1996). Caring and uncaring encounters in nursing and health care. Developing a theory [dissertation]. Linköping; Linköping University; 1996. Medical Dissertation, No 493.

19. Monsma J, Richerson J, Sloand E. Empowering parents for evidence-based fever management: An integrative review. J Am Assoc Nurse Pract. 2015;27(4):222–9. doi: 10.1002/2327-6924.12152

20. Thompson AP, Nesari M, Hartling L, et al. Parents' experiences and information needs related to childhood fever: A systematic review. Patient Educ Couns. 2020;103(4):750-63. doi: 10.1016/j.pec.2019.10.004

For peer review only

21. Westin, E, Sund Levander, M. Parent's Experiences of Their Children Suffering Febrile Seizures. J Pediatr Nurs. 2018;38:68-73. doi: 10.1016/j.pedn.2017.11.001.

22. Eriksson I, Wilhsson M, Blom T, et al. Telephone nurses' strategies for managing difficult calls: A qualitative content analysis. Nurs Open. 2020;7(6):1671-79. doi: 10.1002/nop2.549.

23. Marton F. Phenomenography – describing conceptions of the world around us. Instructional Science, 1981; 10(2):177-200. doi.org/10.1007/BF00132516

24. Larsson S. Qualitative Analysis The example of Phenomenography, in Swedish (Kvalitativ analys exemplet fenomenografi). Lund: Studentlitteratur; 1981

25. Larsson J, Holmström I. Phenomenographic or phenomenological analysis: does it matter? Examples from a study on anaesthesiologists' work. Int J Qual Stud Health Well-being. 2009;2:55-64. doi:10.1080/17482620601068105

26. World Medical Association. WMA Declaration of Helsinki - Ethical principles for medical research involving human subjects. [online]. 2022. https://www.wma.net/policies-post/wma-declaration-of-helsinki-ethical-principles-for-medical-research-involving-human-subjects/ (accessed 12 February 2023).

27. Greenberg ME. A comprehensive model of the process of telephone nursing. Journal of advanced nursing. 2009;65(12):2621-29. doi: 10.1111/j.1365-2648.2009.05132.x.

28. Kai J. Parents' difficulties and information needs in coping with acute illness in preschool children: a qualitative study. BMJ. 1996;313(7063):987-90. doi: 10.1136/bmj.313.7063.987.

29. Gustafsson S, Sävenstedt S, Martinsson J, et al. Need for reassurance in self-care of minor illnesses. J Clin Nurs. 2018;27(5-6):1183-91. doi: 10.1111/jocn.14157.

30. Kuijpers DL, Peeters D, Boom NC, et al. Parental assessment of disease severity in febrile children under 5 years of age: a qualitative study. BMJ Open. 2021;11:e042609. doi: 10.1136/bmjopen-2020-042609.

31. Lass M, Rahr Tatari C, Hoffmann Merrild C, et al. Contact to the out-of-hours service among Danish parents of small children - a qualitative interview study. Scand J Prim Health Care. 2018;36(2):216-23. doi: 10.1080/02813432.2018.1459431.

32. Peetoom KKB, Ploum LJL, Smits JJM, et al. Childhood fever in well-child clinics: a focus group study among doctors and nurses. BMC Health Serv Res 2016;16:240. doi: 10.1186/s12913-016-1488-1.

For peer review only

33. Thompson AP, Le A, Hartling L, et al. Fading Confidence: A Qualitative Exploration of Parents' Experiences Caring for a Febrile Child. J Clin Nurs. 2020;29(5-6):964-73. doi: 10.1111/jocn.15165.

34. Gamst-Jensen H, Frischknecht Christensen E, Lippert F, et al. Self-rated worry is associated with hospital admission in out-of-hours telephone triage - a prospective cohort study. Scand J Trauma Resusc Emerg Med. 2020;28:53. doi: 10.1186/s13049-020-00743-8.

35. Green JM, Spiby H, Hucknall C, et al. Converting policy into care: women's satisfaction with the early labour telephone component of the All Wales Clinical Pathway for Normal Labour. J Adv Nurs. 2012;68(10):2218-28. doi: 10.1111/j.1365-2648.2011.05906.x.

36. Cabral C, Ingram J, Hay AD, et al. "They just say everything's a virus"—Parent's judgment of the credibility of clinician communication in primary care consultations for respiratory tract infections in children: A qualitative study. Patient Educ Couns. 2014;95(2):248-53. doi: 10.1016/j.pec.2014.01.010.

37. Halls A, Van't Hoff C, Little P, et al. Qualitative interview study of parents' perspectives, concerns and experiences of the management of lower respiratory tract infections in children in primary care. BMJ Open. 2017;7:e015701. doi: 10.1136/bmjopen-2016-015701.

38. Marklund B, Ström M, Månsson J, et al. Computer-supported telephone nurse triage: an evaluation of medical quality and costs. J Nurs Manag. 2007;15(2):180-7. doi: 10.1111/j.1365-2834.2007.00659.x.

39. Ernesäter A, Engström M, Holmström I, et al. Incident reporting in nurse-led national telephone triage in Sweden: the reported errors reveal a pattern that needs to be broken. J Telemed Telecare. 2010;16(5):243-7. doi: 10.1258/jtt.2009.090813.

40. McKenna G, Rogers A, Walker S, et al. The influence of personal communities in understanding avoidable emergency department attendance: qualitative study. BMC Health Serv Res. 2020;20:887. doi: 10.1186/s12913-020-05705-5.

41. Berntsson K, Eliasson M, Beckman L. Patient safety when receiving telephone advice in primary care - a Swedish qualitative interview study. BMC Nurs. 2022;21:24. doi: 10.1186/s12912-021-00796-9.

42. Brousseau DC, Nimmer MR, Yunk NL, et al. Nonurgent emergency-department care: analysis of parent and primary physician perspectives. Pediatrics 2011;127(2):e375-81. doi: 10.1542/peds.2010-1723.

43. Hiller MG, Caffery MS, Bégué RE. A Survey About Fever Knowledge, Attitudes, and Practices Among Parents. Clin Pediatr (Phila). 2019;58(6):677-80. doi: 10.1177/0009922819834276.

For peer review only

44. Sundberg A, Wahlberg AC, Zethraeus N, et al. Observational study of the implementation of telephone advice nursing in Sweden: did callers follow recommendations and did the rate of healthcare visits change? BMJ Open. 2021 Aug;11:e051233. doi: 10.1136/bmjopen-2021-051233.

45. Hamideh Kerdar S, Himbert C, Martin DD, et al. Cross-sectional study of parental knowledge, behaviour and anxiety in management of paediatric fever among German parents. BMJ Open. 2021;11:e054742. doi: 10.1136/bmjopen-2021-054742.

46. Gustafsson S, Vikman I, Axelsson K, et al. Self-care for minor illness. Prim Health Care Res Dev. 2015;16:71-8. doi: 10.1017/S1463423613000522.

47. Lincoln YS, Guba EG. Naturalistic inquiry. (1985). Newbury Park, CA: SAGE Publications.

Figure Legend

Figure 1 - Schematic view of the outcome space

For peer review only

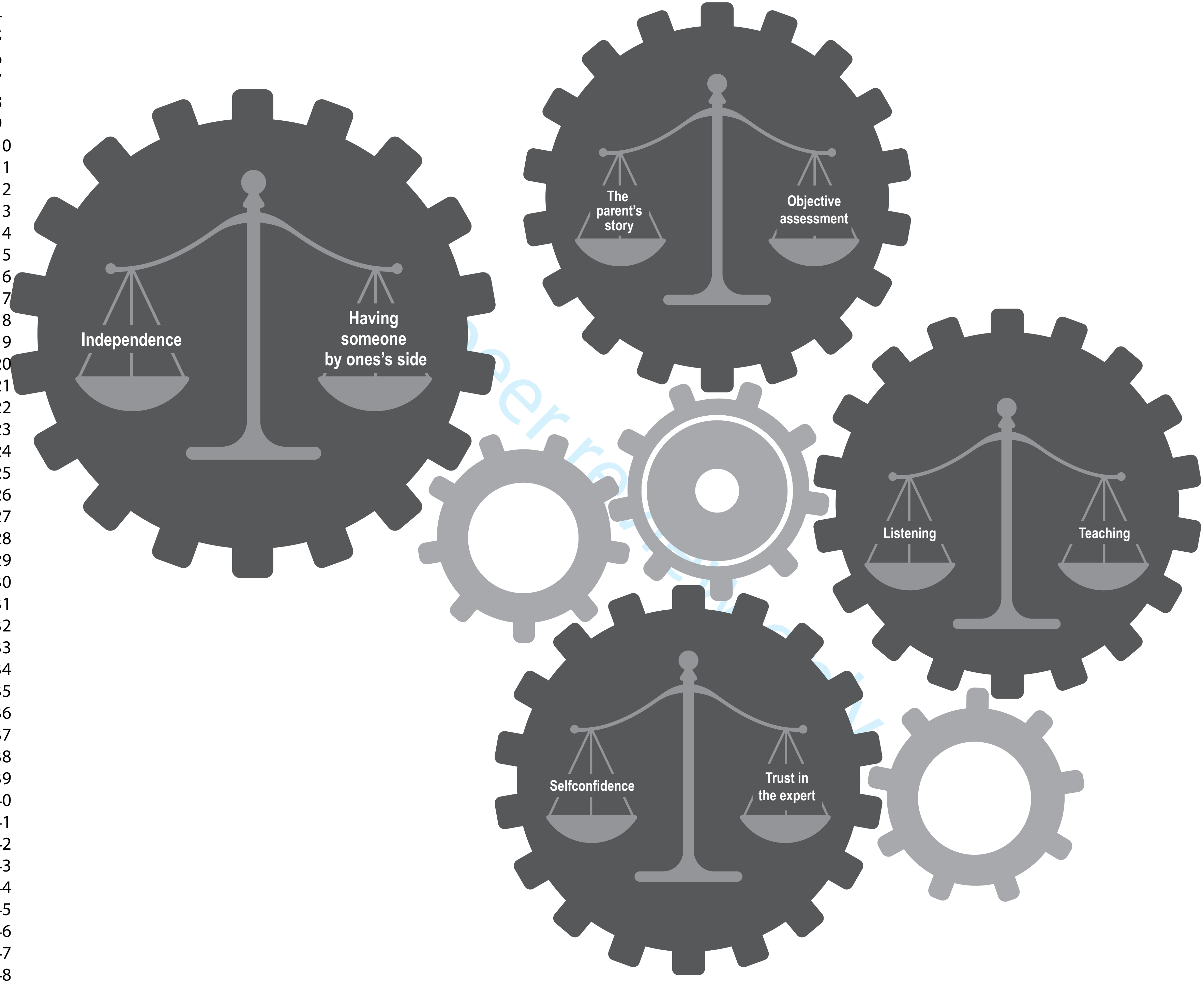
Independence
Having
someone
by ones's side
The
parent's
story
Objective
assessment
Listening
Teaching
Selfconfidence
Trust in
the expert

Interview guide

Background questions

- Which workplace do you have?
- How long have you worked there?
- What education do you have?
- How old are you?

Interview questions

1. **What is advising parents whose child has a fever for you?**

- Tell us how it is done when you give advice to parents whose child has a fever?
- What experiences do you have of your work in advising parents whose children have a fever? Examples of situations perceived as positive/ difficult.
- How do you perceive that you receive support/can be a support to parents whose children have a fever?
- How do you perceive the parents' need for counseling when the child has a fever?
- What opportunities do you see with your consultancy?

Follow up questions like; can you tell me more, what do you mean when you say?

For peer review only

Standards for Reporting Qualitative Research (SRQR)*

http://www.equator-network.org/reporting-guidelines/srqr/

Title and abstract

	Page/line no(s).
**Title** - Concise description of the nature and topic of the study Identifying the study as qualitative or indicating the approach (e.g., ethnography, grounded theory) or data collection methods (e.g., interview, focus group) is recommended	1/1-2
**Abstract** - Summary of key elements of the study using the abstract format of the intended publication; typically includes background, purpose, methods, results, and conclusions	1/4-24

Introduction

**Problem formulation** - Description and significance of the problem/phenomenon studied; review of relevant theory and empirical work; problem statement	2/1-36
**Purpose or research question** - Purpose of the study and specific objectives or questions	2/34-36

Methods

**Qualitative approach and research paradigm** - Qualitative approach (e.g., ethnography, grounded theory, case study, phenomenology, narrative research) and guiding theory if appropriate; identifying the research paradigm (e.g., postpositivist, constructivist/ interpretivist) is also recommended; rationale**	3/ 2-8
**Researcher characteristics and reflexivity** - Researchers' characteristics that may influence the research, including personal attributes, qualifications/experience, relationship with participants, assumptions, and/or presuppositions; potential or actual interaction between researchers' characteristics and the research questions, approach, methods, results, and/or transferability	11/ 2-6, 13-15
**Context** - Setting/site and salient contextual factors; rationale**	1/ 14-28<br>3/ 11-12<br>Table 1
**Sampling strategy** - How and why research participants, documents, or events were selected; criteria for deciding when no further sampling was necessary (e.g., sampling saturation); rationale**	3/ 11-20<br>11/ 7-10
**Ethical issues pertaining to human subjects** - Documentation of approval by an appropriate ethics review board and participant consent, or explanation for lack thereof; other confidentiality and data security issues	3/ 16-18<br>5/ 4-7

For peer review only - http://bmjopen.bmj.com/site/about/guidelines.xhtml

**Data collection methods** - Types of data collected; details of data collection procedures including (as appropriate) start and stop dates of data collection and analysis, iterative process, triangulation of sources/methods, and modification of procedures in response to evolving study findings; rationale**	4/ 2-3
**Data collection instruments and technologies** - Description of instruments (e.g., interview guides, questionnaires) and devices (e.g., audio recorders) used for data collection; if/how the instrument(s) changed over the course of the study	4/ 3-9
**Units of study** - Number and relevant characteristics of participants, documents, or events included in the study; level of participation (could be reported in results)	3/ 18-23<br>Table 1
**Data processing** - Methods for processing data prior to and during analysis, including transcription, data entry, data management and security, verification of data integrity, data coding, and anonymization/de-identification of excerpts	4/ 9-10
**Data analysis** - Process by which inferences, themes, etc., were identified and developed, including the researchers involved in data analysis; usually references a specific paradigm or approach; rationale**	4/ 11-16<br>Box 1<br>Table 2
**Techniques to enhance trustworthiness** - Techniques to enhance trustworthiness and credibility of data analysis (e.g., member checking, audit trail, triangulation); rationale**	10/ 42-43<br>11/ 1-22

Results/findings

**Synthesis and interpretation** - Main findings (e.g., interpretations, inferences, and themes); might include development of a theory or model, or integration with prior research or theory	5/9-15<br>6/1-6, 11-24, 28-34<br>7/1-14, 19-31, 36-39<br>8/1-3, 7-13, 15-23, 29-38<br>Figure 1
**Links to empirical data** - Evidence (e.g., quotes, field notes, text excerpts, photographs) to substantiate analytic findings	6/ 7-10, 25-26, 35-39<br>7/ 15-17, 32-25<br>8/ 4-6, 24-28<br>9/ 1-3

Discussion

**Integration with prior work, implications, transferability, and contribution(s) to the field -** Short summary of main findings; explanation of how findings and conclusions connect to, support, elaborate on, or challenge conclusions of earlier scholarship; discussion of scope of application/generalizability; identification of unique contribution(s) to scholarship in a discipline or field	9/5-41<br>10/1-39
**Limitations** - Trustworthiness and limitations of findings	10/ 42-43<br>11/ 1-22

Other

For peer review only - http://bmjopen.bmj.com/site/about/guidelines.xhtml

**Conflicts of interest** - Potential sources of influence or perceived influence on study conduct and conclusions; how these were managed	12/ 4-5
**Funding** - Sources of funding and other support; role of funders in data collection, interpretation, and reporting	12/ 1-2

*The authors created the SRQR by searching the literature to identify guidelines, reporting standards, and critical appraisal criteria for qualitative research; reviewing the reference lists of retrieved sources; and contacting experts to gain feedback. The SRQR aims to improve the transparency of all aspects of qualitative research by providing clear standards for reporting qualitative research.

**The rationale should briefly discuss the justification for choosing that theory, approach, method, or technique rather than other options available, the assumptions and limitations implicit in those choices, and how those choices influence study conclusions and transferability. As appropriate, the rationale for several items might be discussed together.

Reference:
O'Brien BC, Harris IB, Beckman TJ, Reed DA, Cook DA. **Standards for reporting qualitative research: a synthesis of recommendations.** *Academic Medicine*, Vol. 89, No. 9 / Sept 2014 DOI: 10.1097/ACM.0000000000000388

For peer review only

For peer review only - http://bmjopen.bmj.com/site/about/guidelines.xhtml

BMJ Open

Advising parents when their child has a fever – a phenomenographic analysis of nurses' perceptions when working at a telephone helpline, at primary care or at a children's emergency department in Sweden

Journal:	*BMJ Open*
Manuscript ID	bmjopen-2023-074823.R2
Article Type:	Original research
Date Submitted by the Author:	11-Jan-2024

Complete List of Authors:	Westin, Emma; Linnaeus University - Vaxjo Campus, Department of Health and Caring Sciences ; Region Kronoberg, Department of pediatrics<br>Gustafsson, Ingrid; University College of Boras Faculty of Caring Science Work Life and Social Welfare, Department of caring sciences; Linnaeus University Faculty of Health and Life Sciences, Department of health and caring sciences<br>Svensson, Anders; Linnaeus University Faculty of Health and Life Sciences, Health and Caring Science; Region Kronoberg, Department of Ambulance Service<br>Sund-Levander, Märta; Linkopings universitet, Department of Health and Care<br>Elmqvist, Carina; Linnaeus University Faculty of Health and Life Sciences, Dept. of Health and Caring Sciences, Linnaeus University; Region Kronoberg, Head of Research

**Primary Subject Heading**:	Nursing
Secondary Subject Heading:	Paediatrics, Qualitative research
Keywords:	Nursing Care, PAEDIATRICS, QUALITATIVE RESEARCH

SCHOLARONE™
Manuscripts

I, the Submitting Author has the right to grant and does grant on behalf of all authors of the Work (as defined in the below author licence), an exclusive licence and/or a non-exclusive licence for contributions from authors who are: i) UK Crown employees; ii) where BMJ has agreed a CC-BY licence shall apply, and/or iii) in accordance with the terms applicable for US Federal Government officers or employees acting as part of their official duties; on a worldwide, perpetual, irrevocable, royalty-free basis to BMJ Publishing Group Ltd ("BMJ") its licensees and where the relevant Journal is co-owned by BMJ to the co-owners of the Journal, to publish the Work in this journal and any other BMJ products and to exploit all rights, as set out in our licence.

The Submitting Author accepts and understands that any supply made under these terms is made by BMJ to the Submitting Author unless you are acting as an employee on behalf of your employer or a postgraduate student of an affiliated institution which is paying any applicable article publishing charge ("APC") for Open Access articles. Where the Submitting Author wishes to make the Work available on an Open Access basis (and intends to pay the relevant APC), the terms of reuse of such Open Access shall be governed by a Creative Commons licence – details of these licences and which Creative Commons licence will apply to this Work are set out in our licence referred to above.

Other than as permitted in any relevant BMJ Author's Self Archiving Policies, I confirm this Work has not been accepted for publication elsewhere, is not being considered for publication elsewhere and does not duplicate material already published. I confirm all authors consent to publication of this Work and authorise the granting of this licence.

For peer review only

Advising parents when their child has a fever – a phenomenographic analysis of nurses' perceptions when working at a telephone helpline, at primary care or at a children's emergency department in Sweden

Emma Westin [1]

RN, PN, Doctoral student, Department of Health and Caring Sciences, Linnaeus University Växjö, Department of Pediatrics, Region Kronoberg, Sweden. ORCID: 0000-0001-6284-5859

Ingrid Gustafsson[2, 1]

RNA, PhD, Assistant Professor, Department of Caring Science, University of Boras, Boras, Department of Health and Caring Sciences, Linnaeus University, Växjö, Sweden ORCID: 0000-0002-5932-6078

Anders Svensson [1]

RN, PEN, PhD, Assistant Professor, Department of Health and Caring Sciences, Linnaeus University, Växjö, Department of Ambulance Service, Region Kronoberg, Sweden. ORCID: 0000-0001-7479-8092

Märta Sund-Levander

RNT, PhD, Senior Associate Professor, Medical faculty, Department of Health and Care, Linköping University, Sweden. ORCID: 0000-0002-1281-885X

For peer review only

Carina Elmqvist, [1]

RN, PhD, Professor, Department of Health and Caring Sciences, Linnaeus University, Växjö, Head of Research, Region Kronoberg, Sweden. ORCID: 0000-0001-8376-8805

[1]Centre of Interprofessional Collaboration within Emergency care (CICE), Linnaeus university

[2]Faculty of Caring Science, Work Life and Social Welfare, University of Boras

Corresponding author: Emma Westin emma.westin@lnu.se

For peer review

Abstract

Objectives: To describe nurses' perceptions of advising parents when their child has a fever.

Design/method: Inductive, descriptive study with a qualitative, phenomenographic approach.

Participants and setting: A purposive sampling was used. To be included, the 24 online interviewed nurses had to have experience advising parents of febrile children between birth and five years of age. They were recruited from three different parts of the healthcare system from four regions in the south of Sweden.

Results: The nurses described advising parents when their child has a fever as four different kinds of balancing acts: Balancing between the parents' story and objective assessment, balancing between listening and teaching, balancing between self-confidence and trust in the expert, and balancing between independence and having someone by one's side.

Conclusions: Giving advice to parents when a child has a fever is a process where the nurse needs to listen, assess and give advice based on the situation. This requires a correct assessment that depends on the parents' story. Creating a trusting relationship is perceived as necessary for parents to assimilate the advice that is provided. What dominates are the nurses' perceptions of the inner qualities required to achieve a balance in the process, for example the importance of experience and security in their professional role, while it is also necessary to get support from colleagues.

Strengths and limitations of this study

- Two of the five authors have experience in the phenomenographic method, and all of the authors have experience in qualitative methods.
- The first author carried out and transcribed all the interviews.
- There is great variation in the participants workplace, work experience and age.
- The interviews were done via video-conference call, and although most of the participants were accustomed to that way of communicating, this could limit the participants' ways of expressing themselves.

Introduction

In Sweden, parents can generally seek advice via telephone nursing services or within primary care (1). Advising is defined as helping the parents assess the need for healthcare, give advice and information about measures that the parents can do themselves, and, if necessary, refer to appropriate level of care (2). The telephone helpline 1177 Vårdguiden (henceforth 1177) are accessible around the clock all year round. The main focus for the telenurses at 1177 is to advise on different health problems (3). To support their advice, they use a computerized decision support tool (CDST); the same system is also frequently used by nurses who work in primary care. Primary care centers are responsible for preventing, diagnosing and treating illnesses, and, if necessary, sending referrals to specialist care (4). They must offer services for both planned and unplanned visits in general medicine during the day, as well as some on-call services. Primary care also includes child healthcare (5). The goal of child healthcare is to promote health and prevent illness in children from birth until the child starts school by following the child's development and health status and providing support to parents (6). If the child has acute symptoms, the parents should go directly to the pediatric emergency department, whose main purpose is to provide fast and highly specialized care for acute, sometimes life-threatening conditions. However, it is common for parents to go there for minor illnesses; in those cases, advice from a nurse may sometimes be the only course of action (7).

Feverish children cause concerns and are among the most common reasons why parents contact telephone nursing services (8-10), primary care (11-12) or children's emergency departments (7, 12-15). The most common expectations parents have is to get their child a physical examination by a physician, and to receive reassurance and information about warning signs (16). The initial meeting within the healthcare system is usually with a nurse, who is responsible both for creating a trusting relationship and for providing adequate information (17). According to Halldórsdóttir (18), this first encounter is decisive in whether the meeting and the relationship will be perceived as caring or non-caring (18). It is important that the nurses can identify the parents' needs, which can vary depending on background, expectations and previous experience, in order to decide what strategies that can be used to empower them (19). Previous studies have examined parents' views of having a child with fever, with or without contact with the healthcare system (20, 21). It is also recognized that

For peer review only

nurses can play an important role in providing knowledge and advice to these parents (19). However, previous research shows that advising can be experienced as an advanced and demanding task (22). Knowledge about nurses' perceptions is needed in order to prepare and support nurses taking on this role. This study therefore aims to describe nurses' perceptions of advising parents when their child has a fever, in a Swedish context.

Methods

Design

inductive, descriptive study with a qualitative approach was conducted i.e., phenomenography which describes the different perceptions that exist of a phenomenon. This is referred to by Marton (23) as the second-order perspective. The first-order perspective concerns objective observations, i.e., facts (23). Perceptions, on the other hand, are unspoken, implied and unreflected. It is perceptions that form the frame of reference for our thinking and the basis for our opinions (24).

Participants

Purposive sampling was used to obtain a rich and varied understanding of the phenomenon. That included nurses from four different regions in southern Sweden from three different parts of the healthcare system; the telephone helpline 1177 Vårdguiden; primary healthcare, including child healthcare; and a pediatric emergency department. To be included, the nurses had to have experience advising parents of febrile children between birth and five years of age. The first author contacted the department head at each workplace, who then sent out information about the study to the nurses. Those who then chose to participate contacted the author directly. The participants received both written and oral information about the study and provided informed written consent. They were informed that participation was voluntary and that they had the right to cancel their participation in the study at any time. To find all the different perceptions that can exist in a group, Larsson och Holmström (25) believes that approximately 20 interviews are sufficient, therefor the goal was to get between 6-8 participants from each part of the healthcare system and with a geographical spread. This resulted in a total of 24 participants. The participants varied between 27 and 64 years (average 45), and their experience varied from 11 months to 25 years. Of the 24 nurses, 13 had at least one specialist nurse education, most commonly pediatric nurse or primary healthcare nurse (see Table 1).

Table 1. Demographics of the 24 nurses.

For peer review only

	Telephone helpline 1177 (n=8)	Primary health care central and child health central (n=8)	Pediatric emergency department (n=8)
Gender (F/M)	8/0	8/0	6/2
Age (mean years)	48-64 (55,75)	34-64 (46,38)	27-44 (34,25)
Specialist nurse education (yes/no)	2/6	7/1	4/4
Work experience (mean years)	0,9-15 (5.6)	3,5-25 (8.6)	2,5-10 (7.8)

For peer review only

Data collection

The interviews were conducted via Zoom by the first author during the period from March 2021 to October 2021. Data were collected through semi-structured interviews. The interviews all began with the same open-ended question: "What is advising parents whose child has a fever for you?" Followed by a number of predefined questions according to an interview guide (see supplementary file 1). Follow-up questions such as "Can you tell me more?" and “What do you mean when you say…?” were used in order for the participants to reflect on the phenomenon and deepen their reasoning. Two pilot interviews were conducted, and since they did not lead to any changes in the interview guide, both were included in the study. The interviews lasted between 22 and 51 minutes (average 37 minutes) and were transcribed verbatim. Only the person who conducted the interviews had access to the recorded interviews, i.e., the first author. While the interviews were transcribed, they were de-identified from anything that could reveal the participant's identity. Only those who participated in the analysis had access to the transcriptions.

Data analysis

Data analysis was performed following the seven-steps model of Larsson and Holmström (25) (see Box 1). The analysis is not linear, but rather a back and forth motion between the whole and the parts. The analysis was mainly performed by the first, second and last author, but all authors participated and discussed the various steps during the process. The first, second and last authors started out each step in the analysis together, then the first author finished each step but the second and last authors reflected on and verified the outcome. Regularly scheduled meetings were done with the whole group.

Box 1. The seven steps of data analysis, according to Larsson and Holmström (25).

1. The text of the transcribed interviews was read through repeatedly.
2. The answers to the interview-questions were marked. This second step was done again and again to make sure that the marked text correlated to the questions.
3. In the third step, the authors looked in the marked text for what the focus of the nurse's attention was and how she/he described her/his way of advising parents when their child has a fever. This resulted in a preliminary description of each nurse´s predominant way of understanding their work, in other words, their perceptions.
4. The descriptions was grouped into categories based on similarities and differences. This step was done and redone several times, like a journey between parts and whole to ensure that the categories were found in the material and vice versa.
5. Non-dominant ways of understanding were searched out. For an overview of dominant (++) and non-dominant (+) ways of understanding nurses' perceptions of advising parents when their child has a fever, see Table 2.
6. The hierarchical way the categories were related to one another were defined, structuring the outcome space.
7. Based of outcome space, each category of description was assigned a metaphor, in this case, each category came out as four different kinds of balancing acts.

Table 2. Overview of dominant (++) and non-dominant (+) ways of understanding nurses' perceptions of advising parents when their child has a fever.

Interview	The parents' story and objective assessment	Listening and teaching	Self-confidence and trust in the expert	Independence and having someone by one's side
1	+	+	++	++
2	+	++	++	++
3	+	+	+	++
4	++	+	++	++
5	++	++		+
6	++	++	++	++
7	+	++	+	++
8	++	++	+	++
9	+	++	++	++
10	+	+	+	++
11	+	+	+	+
12	++	+	+	++
13	+	+	+	++
14	+	++	++	++
15	++	+	+	++
16	+	+	++	++
17	++	++	+	++
18	+	+	+	++
19	+	+	+	++
20		+	+	++
21	+		+	+
22	+	+	+	++
23		+	+	+

For peer review only

**24**	+	+	++

Ethical considerations

Ethical approval was granted by the Swedish Ethical Review Authority (Dnr: 2020-03731). In accordance with the Declaration of Helsinki, the integrity and dignity of the participants have been respected in that their data and interviews were treated confidentially (26).

Patient and public involvement

Patients or the public were not involved in the design, conduct, reporting, or dissemination plans of this study.

Results

The nurses perceive advising parents when their child has a fever as four different kinds of balancing acts: Balancing between the parents' story and objective assessment, balancing between listening and teaching, balancing between self-confidence and trust in the expert, and balancing between independence and having someone by one's side. All these balancing acts are part of and influence the process of giving advice, but the balancing between independence and having someone by one´s side was more dominantly expressed than the others according to table 2. For a schematic view of the outcome space, see Figure 1.

Balancing between the parents' story and objective assessment

The nurses perceive advising on fever as a balance between the parents' story and an objective assessment. Not seeing the sick child, as with telenursing, or when the nurse and parent don't speak the same language, is perceived as an additional difficulty. The nurse's objective assessment is based on the parents' subjective interpretation of the child's symptoms and their ability to describe them.

"There, we are also dependent on the parents to actually be able to make an assessment, so they may be dependent on us to get answers, but I am just as dependent on their answers to be able to make my assessment // it's quite interesting actually that it's not just them who need us, but we actually need them, and they know their child best". Q17

For peer review only

The nurses perceive that they are dependent on the parents' stories, but the parents' worries may cause them to assess their children as sicker than they are; at the same time, parents know their children best and their concerns should be taken seriously. This interaction is as a collaboration between nurse and parent where the expertise of the parents concerning their children needs to be respected. The nurses perceive that parents need to provide enough information about the wellbeing of their children for nurses to be able to make an assessment, which requires good communication skills. Worry is perceived as potentially blocking this knowledge and affect the ability of parents to accurately assess their child's fever. The nurses' perceptions are that the degree of worry can be influenced by several different factors such as number of children, personality, culture, resources and previous experiences. The nurse's perceptions are that some parents have experienced a serious event in connection with a fever; others have read scary stories that are shared in the media and on the internet. Parents from countries where serious infections with fever are more common were perceived by the nurses as having greater worry about fever.

"The difficult thing is that there is a lot, there can be a lot of worry and then you don't really get the facts, but it is the worries that comes out". J10

Balancing between listening and teaching

The nurses perceive advising about fever as a balance between listening and teaching. Providing information about fever is considered part of a nurse's job, and the interaction between parents and nurses plays a role in how advice is received. It's not just about teaching general knowledge in case of fever, which is something that is simple in and of itself, but the nurse has to listen to the needs of parents and start from there in order to address their specific concerns.

"It will be to create a trust and then to make a knowledge inventory and then come along, with the information, or the advice that I want to give. If I start by giving advice, I don't think the family sees themselves being seen, then it becomes more dismissive even though it is well-intentioned on my part that I want to help. So it has to happen in the right order there" H8

When time is constrained, advising may become more standardized and less based on individual needs. This is perceived as creating a feeling in the parents that they are not being taken seriously. The nurses also perceive that sometimes consensus regarding what is classified as a serious illness is lacking, but regardless of what their own assessment is, the parents' concerns need to be confirmed so they feel they are being heard. This includes, for example, booking an appointment to see a doctor even if the nurse assesses this as being unnecessary. At the same time, the nurses perceive that the needs and wishes of the parents must be weighed against the resources of the healthcare system. The nurses' perceptions are that worried parents have the right to seek medical care, but there are benefits to consensus

For peer review only

when it comes to, for example, the waiting time at the children's emergency department. In the absence of consensus, the likelihood of parents following such advice is perceived as reduced. In these cases, it is considered likely that the parents will seek additional advice, either at the same place or elsewhere. Taking the parents seriously, confirming their concerns and explaining the basis for the assessment are perceived as tools for achieving consensus.

"It's important not to diminish their worries but to show that you listen to them and that it's okay that they're worried. But you have to explain to them, if it's the case that we're not worried, why not in that case so that they understand our work". E5

Balancing between self-confidence and trust in the expert

The nurses' perceptions are that advising on fever is a balance between self-confidence and trust in the expert. Nurses at the children's emergency department are perceived as having the most expertise advising parents, most often using their own knowledge and local directions as guidance. Nurses in other parts of the healthcare system often described using a computerized decision support tool, CDST. It is considered evidence-based support, mainly for nurses who lack experience, but it is also perceived as requiring some experience to be interpreted correctly. CDST provides a structure and has clear directives when it comes to fever. At the same time, it can be perceived as undifferentiated and limited in more complex cases, and in such cases some nurses place more trust in their own knowledge. The fact that some nurses use CDST and some don't may result in parents receiving different answers depending on where they seek advice. This is perceived as causing confusion and sometimes resulting in contradictions between nurses in different parts of the healthcare system.

"Those who have done this decision support, they are specialists, so it's not someone sitting at home and tinkering with this // then, as I said, you have to have your own experience with you, also as a nurse, a newbie, newly trained nurse would not have been so appropriate perhaps to put... in advising, it is not, it is not so simple, so it is not". N14

The nurses perceive though, that the parents generally trust their advice, even if stories about when care has failed to detect and treat serious illness can reduce trust. The trustworthiness is perceived to increase if several people (or sources) say the same thing, if the assessment involves a physician or if the nurse and the parent have an established relation. Establishing trust via phone poses a greater challenge than meeting someone in person. The nurses perceive themselves as providing the most reliable and evidence-based advice and that parents see them as experts and often trust them over themselves.

"There is no opinion in that [the advice], but that it is actually a science that we rely on, I think it is a support that we are actually nurses that they talk to, it's not just anyone, like a grandmother or their mother" T20

Parents are perceived as having different abilities with regard to seeking knowledge about their child's fever, but it's generally perceived as being difficult to sift through all the available

For peer review only

information and assess what is reliable. The nurses perceive that this information overload makes it difficult for parents to fully trust their own knowledge or that of their relatives. The nurses also expressed that even if the parents have general knowledge of fever, they may need help to assess the child's condition and require confirmation that their knowledge is being applied correctly to the situation.

Balancing between independence and having someone by one's side

The nurses perceive that advising on fever is a balance between independence and having someone by one's side. Both training and experience are required, especially on the phone, because fever can represent both harmless and serious conditions. This is also perceived as involving great responsibility as it requires caring for parents as well as the child. The nurse's perceptions are that they need to feel secure in their professional role and not be distressed by the parents' worries. Independently assessing a child with a fever can be perceived as a challenge, especially if it involves a younger child. With increasing experience and knowledge comes independence, but it is still important to have access to support, to have someone by one's side.

"But I think I have the greatest support of my colleagues, so partly I think, despite having worked for a long time, I think it's quite nice that one of my colleagues, it could be the assistant nurse, has also seen the child and says the same as me, that I'm not worried. They have, after all, are often very experienced, have seen a lot of children. Then I feel even more confident in my assessment and can provide better support". B2

This is also perceived by the nurses as being true for the parents. Parents today, though, are perceived as being fearful of fever, of lacking knowledge and support from their relatives, and instead need to seek this support from the healthcare system. This is by the nurses perceived to be one of the most common reasons for parents to seek advice. Therefore, the nurses perceive that parents need and expect healthcare to be accessible. Providing a plan for how parents should deal with their children having a fever and where to turn when this occurs is perceived by the nurses as a way of increasing the independence of parents, by giving them tools to take control of their situation. This can be combined with a follow-up call, where the nurse can determine whether the parents followed the advice that was given and can evaluate the results. This is perceived by the nurses as them supporting the parents.

"Because then I show that I actually care if the situation has improved or not, if they get the help they need, that someone cares about them and that they are not standing there alone in case something happens". D4

Discussion

This study understands nurses' perceptions of advising parents when their child has a fever as four different but related balancing acts. Listening to the parents, making assessments and offering advice can be considered the foundation of the advice-giving process, but the nurses' perceptions show that personal characteristics and peer support are important parts of the process. Overall, these results share similarities with those of Greenberg (27), who has previously described telephone nursing, however as a process comprising three phases. Phase one includes listening to the caller's story and gathering information. In the second phase, the nurse assesses the problem and determines the proper interventions, sometimes with the support of a colleague. The third phase is called output and consists of different nursing actions such as referring to primary care or offering advice for self-management, reassurance and validation. This also describes personal characteristics of the nurse, for example, experience, as influencing factors of the telenursing process (27).

The nurses in the present study perceive difficulties in balancing between the parents' stories and an objective assessment sometimes as a result of difficulties in communication. Earlier studies show that parents often assess and express symptoms differently than healthcare personnel. For example, parents often start from the view of a child's normal behavior and judge the health of the child in relation to how much the behavior deviates from the usual (28-30). Parents may find it difficult to assess and express specific symptoms such as dehydration and breathing problems if they have not experienced them before (30). Conversely, healthcare personnel usually assess illness based on specific symptoms, which can lead to mistakes in communication (31). Greenberg (27) emphasizes that nurses interpreting between healthcare information and information that the caller can understand serves as an important component that links together different aspects of the telenursing process (27). The nurses in the present study perceived that parents' worry affects the way parents assess the symptoms of their children. Previous studies show that it can be difficult for parents to assess their sick child and know when it is time to seek medical care or when self-care is enough (32-33). Gamst-Jensen et al (34) shows that in telephone triage there is a relationship between parents' self-rated anxiety and hospitalization. The perceptions of the nurses in this study suggest that it is important to take parents' concerns into account whether you talk to the parents on the phone or meet them in person.

The nurses in the present study perceive that there had to be a balance between listening and teaching and that their relationship with the parents would decide whether they could reach them with their advice. Halldórsdóttir (18) argues that in order to achieve a caring relationship, the nurse must appear to genuinely care. The nurse needs to build a bridge where the parent feels a sense of belonging in the meeting. If, on the other hand, the parent feels that the nurse is lacking in caring and doesn't want or can't meet, a wall is built that makes it difficult to communicate and create trust (18). For example, the nurses in the present study described a gatekeeping role; even though the nurses wanted to provide service, they felt limited by the resources of the healthcare system. Being refused access to the desired healthcare can give a feeling of not being taken seriously (29, 35), which can then build a wall between parents and nurses. If there is a disagreement about how serious the child's illness is and the nurse fails to explain her assessment, it can lead to mistrust of the given advice. If the parents are not satisfied with the advice, there is a risk that they will seek advice again in

For peer review only - http://bmjopen.bmj.com/site/about/guidelines.xhtml

the near future (36-37). To some extent, this may be due to the parents failing to regain a sense of control over the situation (28, 35).

The nurses in the present study expressed a perceived hierarchy within the healthcare system with regard to who was considered to be experts. A common reason for parents turning to the pediatric emergency department for a minor illness is that they have a higher level of trust in specialist care (often in combination with poor availability in primary care) (14-16, 22, 38). However, it has also been shown that within the healthcare system there is a large proportion of incorrect referrals to the pediatric emergency department (14, 16, 39-40). This could be related to healthcare personnel unwilling to risk making a wrong referral (41-42). This can strengthen the parents' image that it's in the pediatric emergency department that they will receive the best care and create a search pattern that is inadequate (43). This can also cause contradictions between the different parts of the healthcare system (39). It should be added, however, that the majority of studies on telenursing show that telenurses generally advise seeking a lower level of care than the caller first intended (10, 38, 44).

The nurses in this study had perceptions about who and what information and advice to trust when caring for a febrile child. While stating that parents could find their own information, it was understood that this might be difficult for someone without healthcare education, and the nurses perceived themselves to be the experts. Earlier studies have shown that parents are just as aware as the nurses that it can be difficult to know what information is reliable; however, contrary to the perceptions of the nurses in this study, parents do seek, and get, help from both family and friends, as well as the internet, when they need information and advice (11, 33, 45-46). On the other hand, when information and advice came from healthcare personnel, it had the highest compliance (46), which may be a sign that information from healthcare personnel, as well as by parents is considered to be the most reliable information source.

The nurses in the present study perceive a balance between independence and having someone by one's side. Giving advice on fever is perceived as difficult, and the nurses sometimes need support from colleagues in their decision-making when advising parents on this matter, something that has been seen in previously studies as well, however, these studies have focused solely on telenursing (22, 41). Advising via telephone is even more complicated, because the assessment is usually done through a parent. This type of work requires experience, which may be considered even more important than education (41).

Strengths and limitations

According to Lincoln and Guba (47), the trustworthiness of a qualitative study is founded on four pillars: *dependability, confirmability, credibility* and *transferability* (47). In order to ensure *dependability* and *confirmability*, the authors have tried to provide a clear description on how the study was performed as well as citations to support the results. None of the authors have any type of relationship with the participants. The first author has experience advising parents when a child has a fever but has never worked in any of the participants' workplaces. Together with the research group, the first author continuously reflected on her own pre-understanding to avoid bias. Interviews were performed, since this may be considered the most common tool to collect data for this kind of study (24). Since Larsson och Holmström (25) believes that

For peer review only

approximately 20 interviews are sufficient to find all the different perceptions that can exist in a group, the 24 interviews that were conducted were considered an appropriate amount of data to handle and they were judged to be rich in different perceptions. Due to the Covid-19 pandemic, the interviews were done via video-conference call, and although most of the participants were accustomed to this way of communicating, it could limit the participants' ways of expressing themselves. Investigator triangulation was used to ensure *credibility*. Two of the five authors had experience in the phenomenographic method, and all of the authors had experience in qualitative methods. A particularly large focus was on separating what belonged to first- and second-order perspectives, as this is a fundamental aspect of the phenomenographic analysis. The findings were continuously reflected on until consensus arose. To ensure that the reader can assess *transferability* to their own setting, settings and participants were described thoroughly. There may be differences in the educational and healthcare systems of different countries, though, which should be taken into consideration.

Conclusion and relevance to clinical practice

Giving advice to parents when a child has a fever is a process where the nurse needs to listen, assess and give advice based on the situation. This requires a correct assessment that depends on the parents' story. Creating a trusting relationship is perceived as necessary for parents to assimilate the advice that is provided. What dominates are the nurses' perceptions of the inner qualities required to achieve a balance in the process, for example the importance of experience and security in their professional role, while it is also necessary to get support from colleagues. This study can be used as a foundation for discussion and reflection for nurses who work with this type of advising, but also as teaching material for students. It can also help nurses from different parts of the healthcare system to understand each other's work. Future research should focus on how interactions between nurses and parents affect the outcome of the advice-giving process from the view of both parents and nurses.

Author contributions

All authors (EW, IG, AS, MSL and CE) designed and planned the study together. EW was responsible for the data collection i.e. conducted and transcribed the interviews. The analysis was mainly performed by EW, IG and CE, but all authors participated and discussed the various steps during the process. All authors drafted, edited and finally approved the manuscript.

Competing interests

None

Funding statement

This work was supported by Region Kronoberg grant number 969335.

For peer review only

Data availability statement

De-identified data may be available upon reasonable request via the corresponding author.

References

1. Ellbrant JA, Åkeson J, Karlsland Åkeson P. Influence of awareness and availability of medical alternatives on parents seeking paediatric emergency care. Scand J Public Health. 2018;46(4):456-62. doi: 10.1177/1403494817735222

2. The delegation for increased accessibility in healthcare. The path to increased accessibility – long-term, strategic and in collaboration: interim report, in Swedish (Vägen till ökad tillgänglighet – långsiktig, strategisk och i samverkan: delbetänkande). (SOU 2021:59). [online]. https://www.regeringen.se/contentassets/b4d9ad7b41624ea79e9ff4be8d2cbf80/sou-2021_59.pdf (accessed 12 February 2023).

3. Swedish Healthcare Direct. When you call 1177, in Swedish (När du ringer 1177) [online]. 2023. http://www.1177.se/Om-1177/1177-sjukvardsradgivning/ (accessed 12 January 2023).

4. Anell A. The function, organization and finances of primary care - a literature review: Report to the inquiry A national coordinator for more efficient resource utilization in health care, in Swedish (Primärvårdens funktion, organisation och ekonomi – en litteraturöversikt: Rapport till utredningen En nationell samordnare för effektivare resursutnyttjande inom hälso- och sjukvården). (S 2013:4). Socialdepartementet, 2015. 55 p. (Statens offentliga utredningar).

5. National Board of Health and Welfare. The mission of primary care. A survey of how the county council's mandate for primary care is formulated, in Swedish (Primärvårdens uppdrag. En kartläggning av hur landstingens uppdrag till primärvården är formulerade) [online]. 2016. https://www.socialstyrelsen.se/globalassets/sharepoint-dokument/artikelkatalog/ovrigt/2016-3-2.pdf (accessed 7 February 2023).

6. National Board of Health and Welfare. Guidance for child health care, in Swedish (Vägledning för barnhälsovården) [online]. 2014. https://www.socialstyrelsen.se/globalassets/sharepoint-dokument/artikelkatalog/vagledning/2014-4-5.pdf (accessed 7 February 2023).

7. Ellbrant J, Åkeson J, Karlsland Åkeson, P. Pediatric emergency department management benefits from appropriate early redirection of nonurgent visits. Pediatr Emerg Care 2015;31(2):95-100. doi: 10.1097/PEC.0000000000000348.

For peer review only

8. Gren C, Pedersen MK, Hasselager AB, et al. How parents express their worry in calls to a medical helpline: a mixed methods study. BMC Prim Care. 2022;23:80. doi: 10.1186/s12875-022-01680-4.

9. Kaminsky E, Carlsson M, Höglund AT, et al. Paediatric health calls to Swedish telenurses: a descriptive study of content and outcome. J Telemed Telecare. 2010;16(8):454-7. doi: 10.1258/jtt.2010.100110.

10. Keatinge D, Rawlings K. Outcomes of a nurse-led telephone triage service in Australia. Int J Nurs Pract. 2005;11:5-12. doi: 10.1111/j.1440-172X.2005.00495.x.

11. de Bont EG, Lepot JM, Hendrix DA, et al. Workload and management of childhood fever at general practice out-of-hours care: an observational cohort study. BMJ Open. 2015;5:e007365. doi: 10.1136/bmjopen-2014-007365.

12. Nokoff N, Brunner AM, Linakis JG, et al. Presentation to either the pediatric emergency department or primary care clinic for acute illness: the caregivers' perspective. Pediatr Emerg Care. 2014;30(3):146-50. doi: 10.1097/PEC.0000000000000082.

13. Drent AM, Brousseau DC, Morrison AK. Health Information Preferences of Parents in a Pediatric Emergency Department. Clin Pediatr (Phila). 2018;57(5):519-27. doi: 10.1177/0009922817730346.

14. Kubicek K, Liu D, Beaudin C, et al. A profile of nonurgent emergency department use in an urban pediatric hospital. Pediatr Emerg Care. 2012;28(10):977-84. doi.org/10.1097/pec.0b013e31826c9aab

15. Smith V, Mustafa M, Grafstein E, et al. Factors Influencing the Decision to Attend a Pediatric Emergency Department for Nonemergent Complaints. Pediatr Emerg Care 2015;31(9):640-4. doi: 10.1097/PEC.0000000000000392.

16. O'Cathain A, Connell J, Long J, et al. 'Clinically unnecessary' use of emergency and urgent care: A realist review of patients' decision making. Health Expect. 2020;23:19-40. doi: 10.1111/hex.12995.

17. Swedish Nursing Association. Competence description for registered nurses, in Swedish (Kompetensbeskrivning för sjuksköterskor) [online]. 2017. https://www.swenurse.se/publikationer/kompetensbeskrivning-for-legitimerad-sjukskoterska (accessed 7 February 2023).

For peer review only

18. Halldórsdóttir S. (1996). Caring and uncaring encounters in nursing and health care. Developing a theory [dissertation]. Linköping; Linköping University; 1996. Medical Dissertation, No 493.

19. Monsma J, Richerson J, Sloand E. Empowering parents for evidence-based fever management: An integrative review. J Am Assoc Nurse Pract. 2015;27(4):222–9. doi: 10.1002/2327-6924.12152

20. Thompson AP, Nesari M, Hartling L, et al. Parents' experiences and information needs related to childhood fever: A systematic review. Patient Educ Couns. 2020;103(4):750-63. doi: 10.1016/j.pec.2019.10.004

21. Westin, E, Sund Levander, M. Parent's Experiences of Their Children Suffering Febrile Seizures. J Pediatr Nurs. 2018;38:68-73. doi: 10.1016/j.pedn.2017.11.001.

22. Eriksson I, Wilhsson M, Blom T, et al. Telephone nurses' strategies for managing difficult calls: A qualitative content analysis. Nurs Open. 2020;7(6):1671-79. doi: 10.1002/nop2.549.

23. Marton F. Phenomenography – describing conceptions of the world around us. Instructional Science, 1981; 10(2):177-200. doi.org/10.1007/BF00132516

24. Larsson S. Qualitative Analysis The example of Phenomenography, in Swedish (Kvalitativ analys exemplet fenomenografi). Lund: Studentlitteratur; 1981

25. Larsson J, Holmström I. Phenomenographic or phenomenological analysis: does it matter? Examples from a study on anaesthesiologists' work. Int J Qual Stud Health Well-being. 2009;2:55-64. doi:10.1080/17482620601068105

26. World Medical Association. WMA Declaration of Helsinki - Ethical principles for medical research involving human subjects. [online]. 2022. https://www.wma.net/policies-post/wma-declaration-of-helsinki-ethical-principles-for-medical-research-involving-human-subjects/ (accessed 12 February 2023).

27. Greenberg ME. A comprehensive model of the process of telephone nursing. Journal of advanced nursing. 2009;65(12):2621-29. doi: 10.1111/j.1365-2648.2009.05132.x.

28. Kai J. Parents' difficulties and information needs in coping with acute illness in preschool children: a qualitative study. BMJ. 1996;313(7063):987-90. doi: 10.1136/bmj.313.7063.987.

29. Gustafsson S, Sävenstedt S, Martinsson J, et al. Need for reassurance in self-care of minor illnesses. J Clin Nurs. 2018;27(5-6):1183-91. doi: 10.1111/jocn.14157.

For peer review only

30. Kuijpers DL, Peeters D, Boom NC, et al. Parental assessment of disease severity in febrile children under 5 years of age: a qualitative study. BMJ Open. 2021;11:e042609. doi: 10.1136/bmjopen-2020-042609.

31. Lass M, Rahr Tatari C, Hoffmann Merrild C, et al. Contact to the out-of-hours service among Danish parents of small children - a qualitative interview study. Scand J Prim Health Care. 2018;36(2):216-23. doi: 10.1080/02813432.2018.1459431.

32. Peetoom KKB, Ploum LJL, Smits JJM, et al. Childhood fever in well-child clinics: a focus group study among doctors and nurses. BMC Health Serv Res 2016;16:240. doi: 10.1186/s12913-016-1488-1.

33. Thompson AP, Le A, Hartling L, et al. Fading Confidence: A Qualitative Exploration of Parents' Experiences Caring for a Febrile Child. J Clin Nurs. 2020;29(5-6):964-73. doi: 10.1111/jocn.15165.

34. Gamst-Jensen H, Frischknecht Christensen E, Lippert F, et al. Self-rated worry is associated with hospital admission in out-of-hours telephone triage - a prospective cohort study. Scand J Trauma Resusc Emerg Med. 2020;28:53. doi: 10.1186/s13049-020-00743-8.

35. Green JM, Spiby H, Hucknall C, et al. Converting policy into care: women's satisfaction with the early labour telephone component of the All Wales Clinical Pathway for Normal Labour. J Adv Nurs. 2012;68(10):2218-28. doi: 10.1111/j.1365-2648.2011.05906.x.

36. Cabral C, Ingram J, Hay AD, et al. "They just say everything's a virus"—Parent's judgment of the credibility of clinician communication in primary care consultations for respiratory tract infections in children: A qualitative study. Patient Educ Couns. 2014;95(2):248-53. doi: 10.1016/j.pec.2014.01.010.

37. Halls A, Van't Hoff C, Little P, et al. Qualitative interview study of parents' perspectives, concerns and experiences of the management of lower respiratory tract infections in children in primary care. BMJ Open. 2017;7:e015701. doi: 10.1136/bmjopen-2016-015701.

38. Marklund B, Ström M, Månsson J, et al. Computer-supported telephone nurse triage: an evaluation of medical quality and costs. J Nurs Manag. 2007;15(2):180-7. doi: 10.1111/j.1365-2834.2007.00659.x.

39. Ernesäter A, Engström M, Holmström I, et al. Incident reporting in nurse-led national telephone triage in Sweden: the reported errors reveal a pattern that needs to be broken. J Telemed Telecare. 2010;16(5):243-7. doi: 10.1258/jtt.2009.090813.

40. McKenna G, Rogers A, Walker S, et al. The influence of personal communities in understanding avoidable emergency department attendance: qualitative study. BMC Health Serv Res. 2020;20:887. doi: 10.1186/s12913-020-05705-5.

For peer review only

41. Berntsson K, Eliasson M, Beckman L. Patient safety when receiving telephone advice in primary care - a Swedish qualitative interview study. BMC Nurs. 2022;21:24. doi: 10.1186/s12912-021-00796-9.

42. Brousseau DC, Nimmer MR, Yunk NL, et al. Nonurgent emergency-department care: analysis of parent and primary physician perspectives. Pediatrics 2011;127(2):e375-81. doi: 10.1542/peds.2010-1723.

43. Hiller MG, Caffery MS, Bégué RE. A Survey About Fever Knowledge, Attitudes, and Practices Among Parents. Clin Pediatr (Phila). 2019;58(6):677-80. doi: 10.1177/0009922819834276.

44. Sundberg A, Wahlberg AC, Zethraeus N, et al. Observational study of the implementation of telephone advice nursing in Sweden: did callers follow recommendations and did the rate of healthcare visits change? BMJ Open. 2021 Aug;11:e051233. doi: 10.1136/bmjopen-2021-051233.

45. Hamideh Kerdar S, Himbert C, Martin DD, et al. Cross-sectional study of parental knowledge, behaviour and anxiety in management of paediatric fever among German parents. BMJ Open. 2021;11:e054742. doi: 10.1136/bmjopen-2021-054742.

46. Gustafsson S, Vikman I, Axelsson K, et al. Self-care for minor illness. Prim Health Care Res Dev. 2015;16:71-8. doi: 10.1017/S1463423613000522.

47. Lincoln YS, Guba EG. Naturalistic inquiry. (1985). Newbury Park, CA: SAGE Publications.

Figure Legend

Figure 1 - Schematic view of the outcome space

For peer review only

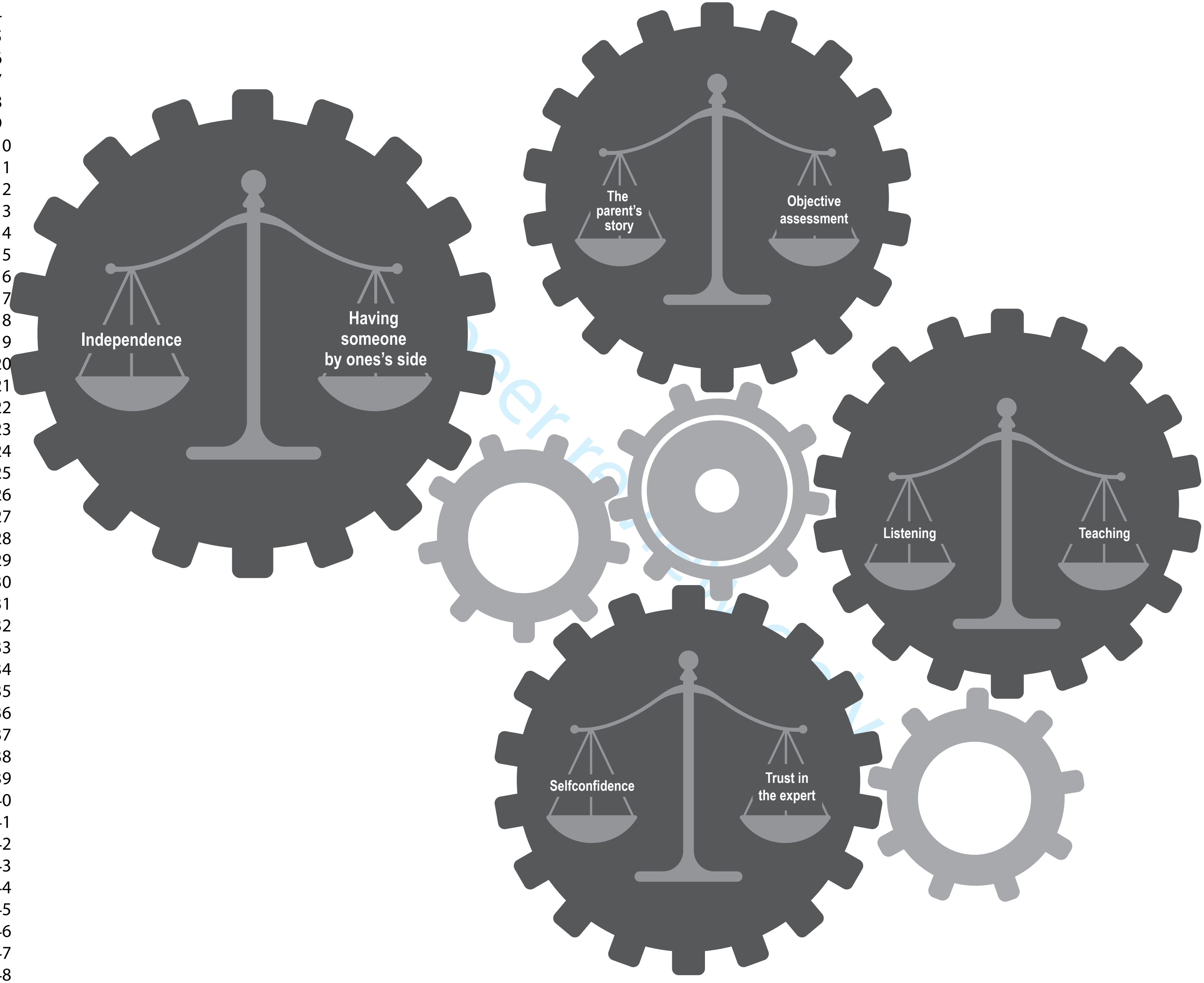
Independence
Having someone by ones's side
The parent's story
Objective assessment
Listening
Teaching
Selfconfidence
Trust in the expert

Interview guide

Background questions

- Which workplace do you have?
- How long have you worked there?
- What education do you have?
- How old are you?

Interview questions

1. **What is advising parents whose child has a fever for you?**

- Tell us how it is done when you give advice to parents whose child has a fever?
- What experiences do you have of your work in advising parents whose children have a fever? Examples of situations perceived as positive/ difficult.
- How do you perceive that you receive support/can be a support to parents whose children have a fever?
- How do you perceive the parents' need for counseling when the child has a fever?
- What opportunities do you see with your consultancy?

Follow up questions like; can you tell me more, what do you mean when you say?

For peer review only

Standards for Reporting Qualitative Research (SRQR)*

http://www.equator-network.org/reporting-guidelines/srqr/

Title and abstract

	Page/line no(s).
**Title** - Concise description of the nature and topic of the study Identifying the study as qualitative or indicating the approach (e.g., ethnography, grounded theory) or data collection methods (e.g., interview, focus group) is recommended	1/1-2
**Abstract** - Summary of key elements of the study using the abstract format of the intended publication; typically includes background, purpose, methods, results, and conclusions	1/4-24

Introduction

**Problem formulation** - Description and significance of the problem/phenomenon studied; review of relevant theory and empirical work; problem statement	2/1-36
**Purpose or research question** - Purpose of the study and specific objectives or questions	2/34-36

Methods

**Qualitative approach and research paradigm** - Qualitative approach (e.g., ethnography, grounded theory, case study, phenomenology, narrative research) and guiding theory if appropriate; identifying the research paradigm (e.g., postpositivist, constructivist/ interpretivist) is also recommended; rationale**	3/ 2-8
**Researcher characteristics and reflexivity** - Researchers' characteristics that may influence the research, including personal attributes, qualifications/experience, relationship with participants, assumptions, and/or presuppositions; potential or actual interaction between researchers' characteristics and the research questions, approach, methods, results, and/or transferability	11/ 2-6, 13-15
**Context** - Setting/site and salient contextual factors; rationale**	1/ 14-28<br>3/ 11-12<br>Table 1
**Sampling strategy** - How and why research participants, documents, or events were selected; criteria for deciding when no further sampling was necessary (e.g., sampling saturation); rationale**	3/ 11-20<br>11/ 7-10
**Ethical issues pertaining to human subjects** - Documentation of approval by an appropriate ethics review board and participant consent, or explanation for lack thereof; other confidentiality and data security issues	3/ 16-18<br>5/ 4-7

For peer review only - http://bmjopen.bmj.com/site/about/guidelines.xhtml

**Data collection methods** - Types of data collected; details of data collection procedures including (as appropriate) start and stop dates of data collection and analysis, iterative process, triangulation of sources/methods, and modification of procedures in response to evolving study findings; rationale**	4/ 2-3
**Data collection instruments and technologies** - Description of instruments (e.g., interview guides, questionnaires) and devices (e.g., audio recorders) used for data collection; if/how the instrument(s) changed over the course of the study	4/ 3-9
**Units of study** - Number and relevant characteristics of participants, documents, or events included in the study; level of participation (could be reported in results)	3/ 18-23<br>Table 1
**Data processing** - Methods for processing data prior to and during analysis, including transcription, data entry, data management and security, verification of data integrity, data coding, and anonymization/de-identification of excerpts	4/ 9-10
**Data analysis** - Process by which inferences, themes, etc., were identified and developed, including the researchers involved in data analysis; usually references a specific paradigm or approach; rationale**	4/ 11-16<br>Box 1<br>Table 2
**Techniques to enhance trustworthiness** - Techniques to enhance trustworthiness and credibility of data analysis (e.g., member checking, audit trail, triangulation); rationale**	10/ 42-43<br>11/ 1-22

Results/findings

**Synthesis and interpretation** - Main findings (e.g., interpretations, inferences, and themes); might include development of a theory or model, or integration with prior research or theory	5/9-15<br>6/1-6, 11-24, 28-34<br>7/1-14, 19-31, 36-39<br>8/1-3, 7-13, 15-23, 29-38<br>Figure 1
**Links to empirical data** - Evidence (e.g., quotes, field notes, text excerpts, photographs) to substantiate analytic findings	6/ 7-10, 25-26, 35-39<br>7/ 15-17, 32-25<br>8/ 4-6, 24-28<br>9/ 1-3

Discussion

**Integration with prior work, implications, transferability, and contribution(s) to the field -** Short summary of main findings; explanation of how findings and conclusions connect to, support, elaborate on, or challenge conclusions of earlier scholarship; discussion of scope of application/generalizability; identification of unique contribution(s) to scholarship in a discipline or field	9/5-41<br>10/1-39
**Limitations** - Trustworthiness and limitations of findings	10/ 42-43<br>11/ 1-22

Other

For peer review only

For peer review only - http://bmjopen.bmj.com/site/about/guidelines.xhtml

**Conflicts of interest** - Potential sources of influence or perceived influence on study conduct and conclusions; how these were managed	12/ 4-5
**Funding** - Sources of funding and other support; role of funders in data collection, interpretation, and reporting	12/ 1-2

*The authors created the SRQR by searching the literature to identify guidelines, reporting standards, and critical appraisal criteria for qualitative research; reviewing the reference lists of retrieved sources; and contacting experts to gain feedback. The SRQR aims to improve the transparency of all aspects of qualitative research by providing clear standards for reporting qualitative research.

**The rationale should briefly discuss the justification for choosing that theory, approach, method, or technique rather than other options available, the assumptions and limitations implicit in those choices, and how those choices influence study conclusions and transferability. As appropriate, the rationale for several items might be discussed together.

Reference:
O'Brien BC, Harris IB, Beckman TJ, Reed DA, Cook DA. **Standards for reporting qualitative research: a synthesis of recommendations.** *Academic Medicine*, Vol. 89, No. 9 / Sept 2014 DOI: 10.1097/ACM.0000000000000388

For peer review only
